# Emergency hospital admissions for stress-related presentations among secondary school-aged minoritised young people in England

**DOI:** 10.1192/bjp.2024.123

**Published:** 2024-11-11

**Authors:** Sorcha Ní Chobhthaigh, Matthew A Jay, Ruth Blackburn

**Affiliations:** 1 UCL, Faculty of Population Health Sciences; 2 UCL GOS Institute of Child Health

**Keywords:** inequality, mental health, stress, adolescent, administrative data

## Abstract

**Background:**

Minoritised young people face a double burden of discrimination through increased risk of stress and differential treatment access. However, acute care pathways for minoritised YP with urgent mental health needs are poorly understood.

**Aims:**

To explore variation in stress-related presentations (SRPs) to acute hospitals across racial–ethnic groups in England.

**Method:**

We examined rates, distribution, duration and types of SRPs across racial-ethnic groups in a retrospective cohort of 11–15-year-olds with 1+ emergency hospital admissions between April 2014 and March 2020. SRPs were defined as emergency admissions for potentially psychosomatic symptoms, self-harm, internalising, externalising, and thought disorders.

**Results:**

White British (8-38 per 1,000 births) and Mixed White-Black (9-42 per 1,000) YP had highest rates of SRPs, while Black African (5-14 per 1,000), Indian (6-19 per 1,000) and White Other (4-19 per 1,000) YP had the lowest. The proportion of YP experiencing repeat admissions were highest for Pakistani (47.7%), White British (41.4%) and Mixed White-Black (41.3%) groups. Black Other (36.4%) and White Other (35.8%) YP had the lowest proportions of re-admissions. YP from Black Other (16.6%), Bangladeshi (16.3%), Asian Other (15.9%), and Black Caribbean (15.8%) groups experienced greater proportions of admissions ≥3 days than their White British (11.9%) and Indian (11.8%) peers. The type of SRPs varied across racial–ethnic groups.

**Conclusions:**

Patterns of SRP admissions systematically differed across racial-ethnic groups, indicative of inequitable triage, assessment and treatment processes. These findings highlight the need for implementation of race equality frameworks to address structural racism in healthcare pathways.

## Introduction

Across ages, ethnicity and race-based discrimination impacts both the risk of mental health difficulties^1^ as well as differential experiences of care. In the UK, differences exist in referral and treatment pathways to mental health care, with racialised and minority ethnic young people (YP) more likely to be referred through social care, criminal justice or secondary care than through primary care, impacting the likelihood of accessing appropriate and timely mental health supports^2,3^. London-based research further indicates that Black and Asian young people are more likely to be referred to inpatient and emergency services, rather than outpatient mental health supports compared to White British young people, indicating differential case management in community settings and primary care^2^. In addition to inequalities in referral pathways and triage, further barriers may impede receipt of treatment and reduce the likelihood of remaining in treatment, including insufficient language translation services, lack of cultural humility, limited treatment options offered, as well as, stereotyping and discrimination perpetuating distrust of professionals^4,5^.

At the extreme, if stress is not adequately managed, it has the potential to escalate, increasing the risk of needing emergency treatment. Previous analyses indicate 7.9% of girls and 4.1% of boys aged 11-17 in England were hospitalised with stress-related presentations^6^. Given the differential pathways and barriers to mental health care for minoritised YP, there is a need to understand their experiences of care seeking and hospital admission at the point of crisis. Moreover, existing inequalities research on mental health care use and referral pathways has largely focused on internalising symptoms, such as, anxiety or depression, despite known variations in manifestations of stress among YP, including emotional, behavioural and physical symptoms. As such, considering the full spectrum of stress-related presentations may provide greater insight into potential bias in pathways and differential access to care.

### Aims

We aimed to explore whether there are systematic differences based on race and ethnicity in hospital admissions for stress-related presentations (SRPs) in secondary school-aged young people. This research addresses the evidence gap examining stress experienced by and healthcare use among minoritised young people in the UK. The project aimed to examine rates of unplanned admissions and indicators of admission experience, including the distribution of repeat admissions, duration of admissions and types of SRPs across racial-ethnic groups. We also examined variation based on assigned sex, in an attempt to understand the complex intersectional experience of gender and racialisation^7^. Given these outcomes reflect those SRPs sufficiently severe that they resulted in an emergency hospital admission, we examined the experience of those during a period of crisis, which may provide insight into differential pathways or missed opportunities for intervention across groups.

## Method

### Data & Population

We used the Hospital Episode Statistics (HES) Admitted Patient Care (APC) dataset, an administrative hospital database capturing all admissions to National Health Service (NHS) hospitals in England^8^. HES APC records information on up to 20 ICD-10 diagnostic codes and 24 operation procedure codes (OPCS-4) for each episode of care recorded by trained clinical coders. Secondary school-age young people aged 11-15 years residing in England who had an emergency (unplanned) hospital admission between 1 April 2014 and 15 March 2020 were eligible for study inclusion (Supplementary Figure 1). We selected the age range 11-15 years to reduce differences in referral pathways as YP in England can choose to leave secondary school for alternative education or training from age 16^9^.

We adopted a birth cohort approach to enable us to examine the ethnic groups as used in the UK Census described below. Disaggregation of racial-ethnic groups is crucial to understanding health inequalities, given the role of differing socio-political-historical factors on health and well-being. ONS mid-year population estimates by year of age were not available according to these groups; nor did we use EthPOP denominators for the same reason.

Given the focus on ethnicity and to allow for exploration of any gender differences, individuals with missing data for assigned sex and ethnicity were excluded from the analyses. Individuals with a recorded death in ONS death registrations were also excluded from the analyses, as their use of health services is likely to differ from other YP.

### Exposure

Our exposure was racial-ethnic group, recognising YP’s recorded ‘ethnicity’ as a proxy for the lived experiences of individuals from minoritized ethnic groups living in a White-British majority country. We intentionally use the term racial-ethnic group throughout this paper given the variable ‘ethnicity’ in UK administrative data includes both ‘race-based’ (“White”) and ‘ethnicity-based’ (“Pakistani”) identifiers. Racial-ethnic groups were coded into 12 groups, merging some groups due to small numbers: White British; White Other (Irish and White Other); Mixed White-Black (White and Black African and White and Black Caribbean); Mixed Other (White and Asian and Mixed Other); Indian; Pakistani; Bangladeshi; Asian Other (Chinese and Asian Other); Black Caribbean; Black African; Black Other; and Other (any other ethnic group). Ethnicity values were obtained across all of YP’s HES APC records; where values conflicted, a modal value was used. If a YP had missing ethnicity across all their APC record, we also sought ethnicity in their HES outpatients and Birth records.

### Outcomes

Outcomes were emergency admission SRPs in HES APC. All emergency admissions in HES APC, regardless of specialty, were included in the analyses. Hospital transfers or admissions within 1 day of discharge were treated as a continuation of the previous admission. An emergency admission was deemed an SRP if the primary diagnosis related to potentially psychosomatic symptoms (e.g. pain, fatigue), mental health problems including internalising, externalising or thought disorders (Supplementary Table 1). Additionally, an admission was counted as an SRP if self-harm was indicated in any diagnostic position. We used the code-list developed by Blackburn et al^8^, which we adapted in line with ICD-11 and to reflect research suggesting that rather than traditional psychiatric classification systems, mental health disorders (encompassed by general psychopathology) are better captured by the two broad latent dimensions, *internalizing* and *externalizing*, as well as a third dimension, *thought disorder*, which captures symptoms of psychosis and mania^10,11,12^(Appendix Table 1). In addition, to exclude admissions where there was a known medical cause, we expanded the list of exclusion codes indicating a medical or surgical cause for physical health symptoms (Appendix Table 2). We excluded SRPs identified in pregnancy-related admissions, defined in line with previous publications^13^.

To capture the complexity and comorbidity that arises in presentations, admissions that included an SRP in the primary diagnostic position and a different SRP code in any other diagnostic position were classified as both. For example, if young person had a primary diagnosis of “F10 - Mental and behavioural disorders due to use of alcohol” and a diagnosis of “F323 - Severe depression with psychotic symptoms” in any other diagnostic position, this would be counted as (1) externalising and (2) thought disorder related admission.

### Cohort Characteristics

HES APC documents patient ‘sex’ and age as well as region and area-level deprivation (the English indices of deprivation)^14^. Given unclear documentation and variation in clinical practice, variable ‘sex’ in patient history was deemed most likely indicative of ‘assigned sex’ at birth or clinician-determined based on gender presentation, as such reference to ‘gender’ or ‘sex’ in this paper refers ‘assigned sex’ not ‘gender identity’ which is not yet reliably captured in HES APC data.

### Analyses

Rates of SRPs across racial-ethnic groups were calculated per 1,000, stratified by assigned sex and age, using HES birth records as a population estimate. We calculated the age-specific incidence of a first SRP and estimated the cumulative incidence of SRP admissions for young people born in 2003-04. The proportion of admissions that were stress-related, as well as the types of SRPs diagnosed during admission and the durations of admissions were compared across racial-ethnic groups. We also compared the proportion of young people with a repeat admission across groups. We calculated the Relative Risk of repeat admissions between 1 April 2014 and 15 March 2020, longer admission duration (≥3 days), types of SRPs and multiple diagnoses during an admission across racial-ethnic groups compared to all groups combined^15^. All data cleaning, preparation and analyses were completed using R for windows version 4.3.0.

Using the available administrative data, it was determined that it is not possible to disentangle the effects of socio-economic position (SEP) from historical and ongoing experiences of discrimination, oppression, and exclusion, in particular, based on class, race, or migration status, which have contributed to lower socio-economic positioning for minoritised groups and directly impacted health outcomes. As such, SEP is on the same causal pathway and adjusting for it risks diluting the impact of systemic inequalities on the health outcomes of interest. Therefore, we chose not to adjust for socio-economic position and report unadjusted results only.

### Ethics statement

This study uses de-identified NHS Hospital Episode Statistics data provided to the researchers by NHS England within the terms of a data-sharing agreement (DARS-NIC-393510-D6H1D-v8.10). Study-specific ethical approval was not required.

## Results

### Summary of cohort and admissions

We identified 210,973 young people aged 11-15 years born in England between 1 April 2003 and 15 March 2009 who had 312,362 emergency admissions between 1 April 2014 and 15 March 2020. Their characteristics are presented in Supplementary Tables 2 and 3.

Combined, 30.9 per cent of all emergency admissions (96,484 of 312,363) were stress-related (Supplementary Table 4). This proportion was highest for White British (32.5%) and lowest for Black African (20.6%) young people.

### Rates of SRP admissions

**Figure 1:**
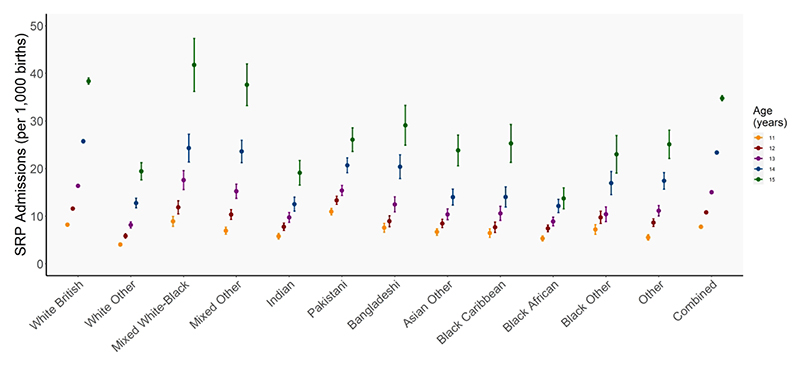
Rates of admission for SRPs per 1,000 births for young people aged 11-15 years born April 2003-March 2009 admitted between April 2014-March 2020 across racial-ethnic groups

Rates of admissions varied by age and ethnic group (Figure 1). At ages 11-13, Pakistani, Mixed White-Black race/ethnicity and White British young people had the highest rates of SRP admissions. At ages 14 and 15, Mixed White-Black race/ethnicity and White British young people continue to experience the highest admission rates, with higher rates among their Mixed Other race/ethnicity peers, as the rate slows for their Pakistani peers. Across all ages, White Other, Black African and Indian young people are among the lowest admission rates.

### Cumulative incidence

By 2019-2020, of the young people born between April 2003-March 2004, 21,436 had been admitted at least once with a SRP. We estimated the cumulative incidence of young people between 11 and 15 years born in England between April 2003-March 2004, to be 5%, highest for Pakistani (5.4%) and White British (5.3%) young people and lowest for Black African (3.0%) and Asian Other (3.2%) young people (Supplementary Figure 2 and Table 6).

### Trends in SRP admissions

**Figure 2:**
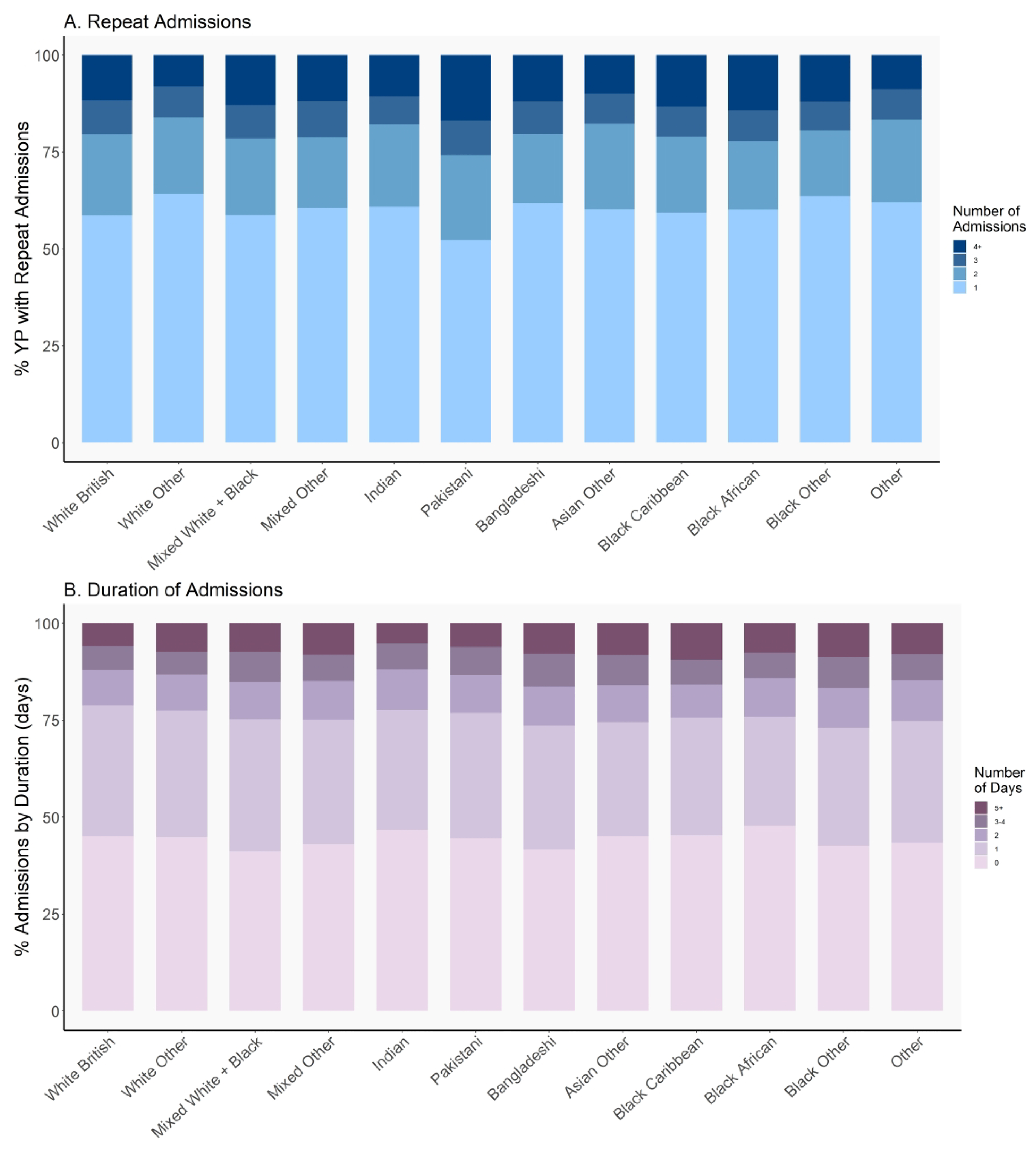
(A) Trends in the percentage of young people admitted with SRP with singular and repeat SRP admissions across racial-ethnic groups (B) Trends in the percentage of SRP admissions by duration of admission in days across racial-ethnic groups

The highest proportion of repeat (2+) admissions were among Pakistani (47.7%), White British (41.4%), and Mixed White-Black race/ethnicity (41.3%) young people, whereas this was less for other minoritised groups, with White Other (35.8%) and Black Other (36.4%) young people least likely to be re-admitted (Figure 2 (a)).

Young people from Black Other (16.6%), Bangladeshi (16.3%), Asian Other (15.9%), and Black Caribbean (15.8%) groups experienced a greater proportion of longer admissions (3 days or more) compared to their White British (11.9%) and Indian (11.8%) peers (Figure 2(b)). Notably, while Bangladeshi and Black Other young people have a correspondingly lower proportion of admissions that lasted less than 1 day (41.7% and 42.7% respectively), Black Caribbean and Asian Other young people are also among the highest proportion of admissions <1 day (45.3% and 45.1% respectively), indicating a differential spread in duration of admissions across racial-ethnic groups.

### Types of SRP admissions

**Figure 3:**
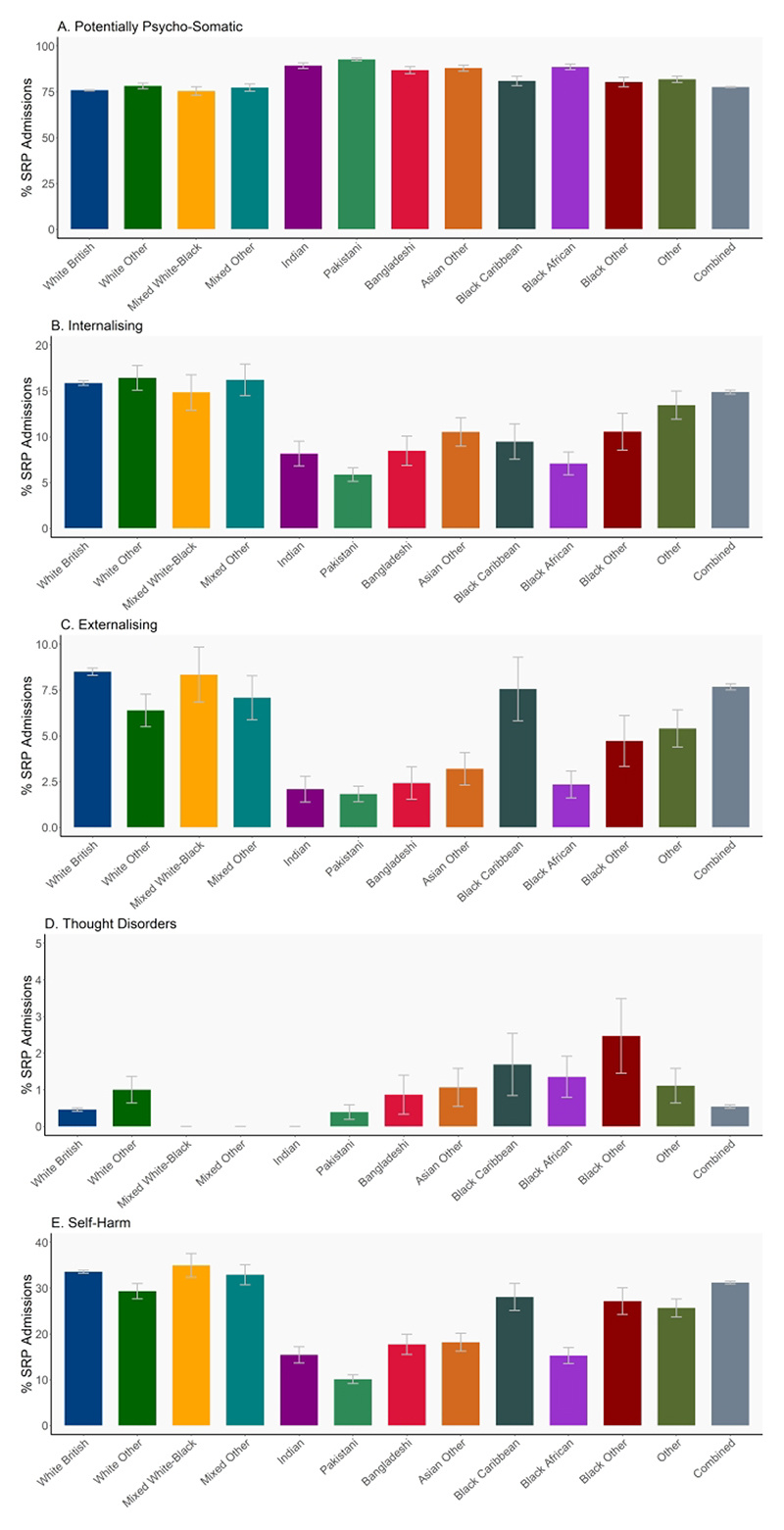
Percentage of SRP Admissions with a recorded diagnoses related to (A) Psycho-Somatic (B) Internalising (C) Externalising (D) Thought disorder* (E) Self-harm symptoms across racial-ethnic groups. Note that y axis scales differ. *Results for Mixed White-Black race/ethnicity, Mixed Other race/ethnicity and Indian groups with recorded diagnoses related to Thought disorder censored due to small cell count.

Patterns for types of SRPs were mixed (Figure 3). Young people from all Asian groups (Indian, Pakistani, Bangladeshi, Asian Other), as well as Black African and Other race/ethnicity groupings were admitted with potentially psycho-somatic symptoms more frequently than their White and Mixed race/ethnicity peers. The opposite trend was found for internalising and self-harm presentations. White and Mixed racial-ethnic groups were admitted most frequently with internalising and self-harm concerns, while Pakistani, Black African and Indian young people were least likely to be recorded with internalising or self-harm diagnoses. Similarly, White British and Mixed race/ethnicity young people were more frequently admitted with externalising concerns, while all Asian groups, as well as Black African young people were admitted least frequently. Young people from all Black groups, Black Other (2.5%), Black Caribbean (1.7%), and Black African (1.4%), were more likely to be diagnosed with thought disorder diagnoses on emergency admission, than their White British (0.5%), Mixed race/ethnicity, Indian and Pakistani (0.4%) peers.

### Multiple diagnoses during admissions

**Figure 4:**
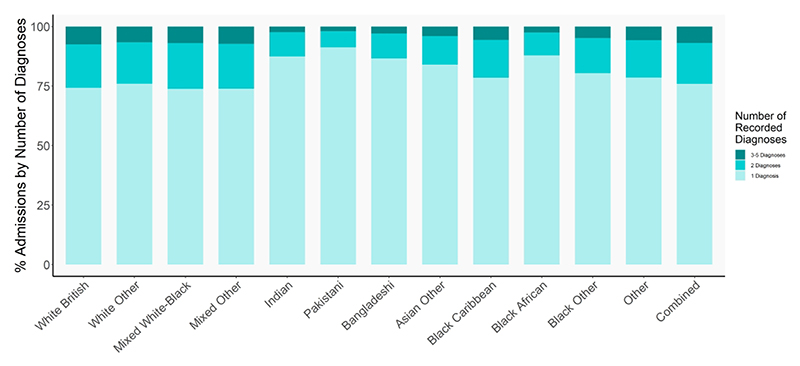
Trends in the percentage of SRP admissions by number of diagnoses recorded during an admission across racial-ethnic groups

During the same SRP admission, young people from White and Mixed race/ethnicity groups were more likely to be diagnosed with more than one type of stress-related presentation than their South Asian (Indian, Pakistani, Bangladeshi) and Black African peers (Figure 4).

### SRP Admissions Visual Summary

The heatmap (Supplementary Figure 3 and Supplementary Table 11) displays the relative risk of each racial-ethnic group (compared to all groups combined) experiencing (1) repeat admissions (2) admission durations ≥3 days, as well as admissions with recorded diagnoses related to (3) Psycho-Somatic (4) Internalising (5) Externalising (6) Self-harm symptoms, and (7) admissions with >1 SRP-related diagnoses during an admission. The relative risk of admissions associated with thought disorder diagnoses is visualised (Supplementary Figure 4 and Supplementary Table 12) for racial-ethnic groups with sufficient cell counts (≥10).

### Intersection with Gender

Across racial-ethnic groups females were more likely to be admitted for stress-related concerns, than their male counterparts (Supplementary Table 5). Across racial-ethnic groups, the gap between female and male admissions widened steadily with age, however the extent varied considerably, with White British, Mixed White-Black, Mixed Other and Other race/ethnicity young people experiencing the widest gap from age 13 onwards. For the 2003-2004 cohort, among females, the cumulative incidence at age 15 was highest for White British (7.1%), lowest for Black African (3.7%) and Asian Other (3.9%). Among males, the cumulative incidence was highest for Pakistani (4.5%) and lowest for Black African (2.4%) and Other race/ethnicity males (2.4%) (Supplementary Table 6).

There were also differences in re-admissions, duration in hospital, types of presentation and multimorbidity (Supplementary Tables 7 to 10). Across groups, males were admitted with somatic symptoms at a higher frequency than females. Pakistani (96.8%), followed by Indian (95.4%) males experienced the highest proportion of admissions related to psychosomatic presentations, while White British females (69.9%) were least likely to be recorded with a psychosomatic-related admission (Supplementary Table 9). The opposite trend was true for admissions associated with internalising and self-harm diagnoses; females were admitted at a higher frequency than males across groups. Females from White and Mixed race/ethnicity groups were admitted with internalising (17.5-20.7%) and self-harm (38.4-43.9%) presentations most frequently, while Pakistani males were least likely to be admitted with either internalising (3.4%) or self-harm (3.2%) concerns (Supplementary Table 9).

## Discussion

### Main findings

Our study is the first to report on rates of mental health related acute hospital admissions for minoritised young people. We identified important variations in admission patterns across racial-ethnic groups, indicative of differential experiences during triage, assessment, admission thresholds and treatment.

Young people from White British, Mixed White-Black and Other race/ethnicity groups experienced the highest overall admission rates, with White Other and Black African young people having the lowest admission rates. These overall admission rates mask more nuanced differences in the proportions of young people who are ever admitted with a stress-related presentation aged 11-15 years (i.e. cumulative incidence) versus those with higher rates of repeat admissions and/or longer durations of hospital stay. Females were more likely to be admitted with stress-related presentations, experience repeat admissions, and remain admitted for longer (relative to males), with the smallest gender gap observed for Indian, Pakistani and Black African young people.

We found variation in the types of stress-related presentations young people from different racial-ethnic groups were admitted with. All Asian groups as well as Black African young people were admitted more frequently with potentially psycho-somatic symptoms and less frequently with internalising, externalising or self-harm presentations, relative to the White and Mixed race/ethnicity groups. Black Caribbean young people were less likely to be admitted with internalising symptoms than their White and Mixed race/ethnicity peers but had similar frequency of externalising and self-harm admissions. Black Other young people were markedly more likely to be diagnosed with a thought disorder during an emergency hospital admission than their Mixed race/ethnicity, Indian, Pakistani and White British peers (2.5% vs <1%).

### Findings in context

#### Inequalities in access

Previous research documents inequalities in accessing timely and appropriate mental health care as well as differential treatment experiences for minoritised young people in the UK^2,3,16^. Given the additional barriers to accessing mental health services, and greater likelihood of referrals to inpatient services minoritised young people may be forced to access emergency services at a point of crisis. Our study of mental health related emergency hospital admissions identified distinct patterns of admissions across racial ethnic groups, painting a nuanced picture. At the extremes, White British young people were among the highest levels of readmissions and lowest levels of extended admissions, the opposite was true for Black Other young people. Although White Other young people were least likely to be re-admitted and Pakistani young people were most likely to be re-admitted, they were equally likely to remain in hospital for admissions of 3 days or more. While Mixed White-Black race/ethnicity, Black Caribbean and Asian Other young people experienced similar levels of readmissions as their White British peers, they continued to experience longer admissions. Notably, not only were Black Caribbean and Asian Other young people more likely to experience longer admissions, they were also among the groups with the highest levels of short admissions (<1 day). It is important to consider that while longer durations may suggest more severe presentations and shorter durations may infer presentations better supported by other services, these variations may also indicate disparities in the perceived relative severity of conditions based on racial-ethnic group. Collectively, these findings indicate structural barriers and potential bias in accessing care that may be driven by inconsistencies in clinical judgement during triage and initial assessment processes, similar to US settings^17,18^.

#### Inequalities in assessment

Where assessments do not account for variations in articulation or conceptualisation of symptoms cross-culturally^19^, symptoms may be more or less likely to be missed, misclassified or receive further assessment, resulting in variation in treatment options offered, admission duration as well as diagnoses recorded. We found that young people racialised as Black or Asian were more likely to be admitted with somatic symptoms than their White British peers. As with all mental health presentations, perceptions of discrimination and microaggressions are associated with increased somatic symptoms, however, findings are mixed regarding whether minoritised individuals are more likely to endorse depression-related somatic symptoms than White individuals^20,21^. Ultimately, it may be that minoritised young people experience differential or culturally insensitive assessment, resulting in physiological concerns being mis-diagnosed as psycho-somatic or alternative presentations being missed or receiving inadequate attention.

Young people from White and Mixed racial-ethnic groups were more likely to be admitted with internalising symptoms, with South Asian groups and Black African young people least likely to be diagnosed with the same. While this is consistent with results of the Mental Health of Children and Young People (MHCYP) in England Survey^22^ which found that anxiety and depressive disorders were most commonly diagnosed in White British and Mixed/Other 5–19-year-olds, it contradicts self-reported difficulties^23^. Among adults, prevalence estimates of internalising mental health conditions vary based on measure, that is, self-report, clinical interview, or professionally diagnosed^24^, while further research is needed to understand variations across racial-ethnic groups among children and young people, in context, these results continue to raise questions about differential assessment procedures and treatment pathways.

Pathways may also be different for externalising symptoms, including impulsivity, aggression and substance-use, which have received less attention in the UK. White British and Mixed-race young people were more likely to be admitted with externalising concerns than their peers, mirroring clinical interview outcomes from the MHCYP in England Survey^22^. Notably, due to the reductive nature of the racial-ethnic group categories used in the MHCYP in England Survey, it is not clear whether our findings mirror the experiences of Black Caribbean young people who were assessed. However, our findings are in line with self-reported difficulties, indicating that Black Caribbean young people were more likely to report experiencing externalizing symptoms^23^. It may be worth considering whether externalising difficulties displayed by White British and Mixed-race young people are more likely to be recognised as indicative of mental health concern, whereas, young people racialised as Black or South Asian, particularly boys, are more likely to be criminalised in UK society^25,26^. This aligns with inequalities in referral pathways showing Black and Asian young people are more likely to be referred to mental health services through justice and social care pathways, rather than directly to health.

Black Other, Black Caribbean, and Black African, young people were more likely to receive diagnoses related to mania and psychosis, on emergency admission, than their White British, Mixed race/ethnicity, Indian and Pakistani peers. In particular, Black Other young people were over 5 times more likely to be diagnosed with thought disorder presentation than White British or Mixed race/ethnicity young people. Diagnosis of psychotic disorders in people under 18 years are considered relatively uncommon (0.4-1.7%) and should only be made after long-term monitoring, referral and comprehensive assessment by specialist mental health service teams^27^. Failure to account for developmental or cultural factors increases the risk of misdiagnosis^28^. Nevertheless, these results reflect the decades long disproportionate diagnoses of psychosis in Black adults in the UK^29^, an increased risk attributed to social and economic disadvantage, structural and interpersonal racism^30,31^.

Consistent with previous UK-based findings, we found all Asian groups and Black African young people were less likely to be admitted for self-harm^32^. As self-harm is an expression of or an attempt to cope with distress rather than a mental health diagnosis, assessment needs to prioritise understanding the function of the behaviour, carefully consider the underlying mental health difficulties in treatment planning^33^. Further, it is well established that a fraction of young people who self-harm actually present to healthcare services as such estimations based on hospital records are likely underestimations of lived experience^34^. Given that Asian and Black African young people were also less likely to receive a secondary diagnosis, taken together, in the context of self-harm, there is a potential for missed indicators of risk, impeding safety and treatment planning.

Further consideration should be given to the ripple effects of the pandemic and school closures, including increased admissions for stress-related presentations^35^, internalising symptoms and rates of self-harm among girls^36^, particularly in the context of widening inequalities.

#### Strengths and limitations

Study strengths include longitudinal and whole nation coverage of acute hospital admissions for young people in England. We refined a previous code-list^6^ (updated in line with ICD-11) to include the full spectrum of stress-related presentations. However, given this broad and complex definition, misclassification is a concern, including instances where a surgical, medical or physiological cause was present but not recorded. We used a Birth Cohort approach to increase specificity in measurement of racial-ethnic groups, however, this resulted in the exclusion of young people not born in England. The relative distributions of racial-ethnic groups in the admissions cohort and Birth Cohort (our population level estimate) were generally consistent, except for White Other and Black African young people who are underrepresented. The mechanism is unclear but may reflect data collection errors including clinician-prescribed racial-ethnic group based on physical characteristics, assumptions based on accent or name. Hospital recorded ethnicity has not been validated and there are conceptual limitations in generalising experiences across racial-ethnic groups as this fails to acknowledge the variations in individuals' lived experiences.

#### Implications & Conclusions

Discussions around barriers to care often centre on stigma and help-seeking behaviours without acknowledging systemic bias and a history of institutionalisation disproportionately experienced by racialised and minoritised communities in the UK. Moreover, it is important to recognise stigma as a learned social response based on historical experience with the function of protecting an individual or group from a negative outcome. By attributing inequalities in access to individual level factors, it overlooks the structural determinants of mental health and deflects from the differential treatment experienced by those who do access services. Our findings indicate differential experiences based on racial-ethnic group in access to care, experiences of admission and documented diagnoses which may reflect underlying structural racism in triage, assessment and treatment processes.

It is therefore imperative that anti-racism practices, such as the NHS England Patient and Carer Race Equality Framework^37^, be implemented across healthcare settings. Clinicians need to be mindful of differences in how individuals express and make sense of their symptoms as well as assumptions and potential biases while making clinical judgements, practicing cultural humility^19,38^. Finally, we need better recording of ‘ethnicity’ in hospital admission records, reflective of an individuals’ self-identified racial-ethnic group. Further research should explore the referral and admission journey from the community to emergency admission as well as trends in planned admissions to inpatient services and variations in discharge and referrals to outpatient services. Also warranted is additional exploration of variation in admission duration and the use of involuntary detainment among young people, which is known to disproportionately affect Black adults in the UK^39^.

## Supplementary Material

Supplementary Materials

## Data Availability

This study uses NHS Hospital Episode Statistics data, provided under the data sharing agreement (DARS-NIC-393510-D6H1D-v8.10) to the researchers by NHS England. The data do not belong to the authors and cannot be shared by the authors, except in aggregate form for publication. The data is collected by the NHS as part of their care and support. Data can be requested through the NHS Digital Data Access Request Service.
