# Supplementary material for: Emergency hospital admissions for stress-related presentations among secondary school-aged minoritised young people in England
Source: Br J Psychiatry. Author manuscript; Available in PMC 2024 Dec 7. (PMC7617073; doi:10.1192/bjp.2024.123)
Supplement: Supplementary Materials [file EMS198182-supplement-Supplementary_Materials.pdf]

Supplementary Figure 1 – Data Flow Diagram

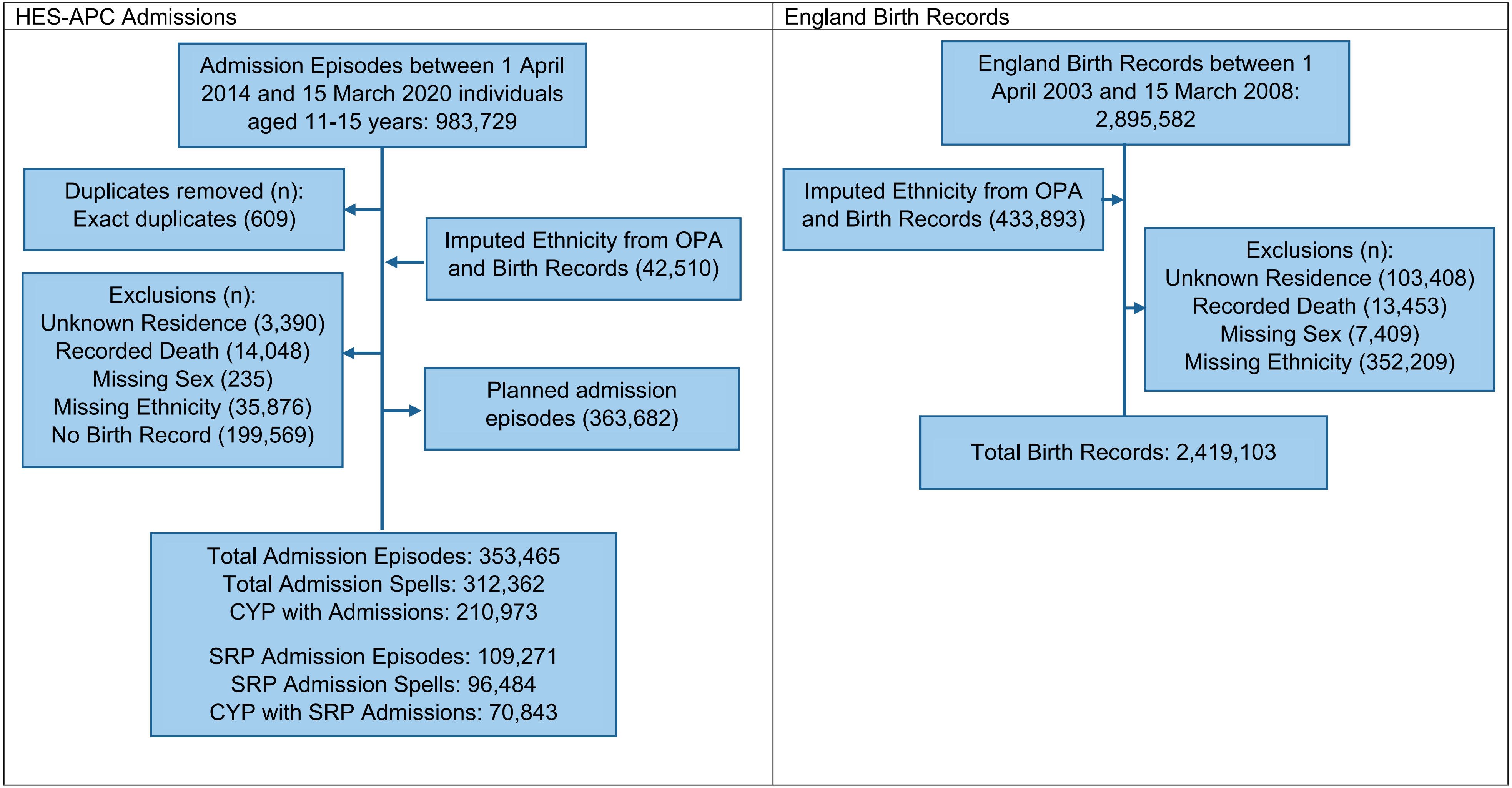

Supplementary Table 1 – Characteristics of young people born in England between 1 April 2003 and 15 March 2008. *Note all numbers rounded to nearest 5.*

n (%)	White British	White Other	Mixed White-Black	Mixed Other	Indian	Pakistani	Bangladeshi	Asian Other	Black Caribbean	Black African	Black Other	Other	All
**Total**	1742180 (72.0)	149300 (6.2)	30545 (1.3)	48180 (2.0)	64125 (2.7)	91695 (3.8)	33685 (1.4)	55140 (2.3)	30105 (1.2)	76360 (3.2)	27085 (1.1)	70700 (2.9)	2419105 (100.0)
**Female**	841770 (48.3)	72310 (48.4)	14860 (48.7)	23110 (48.0)	30630 (47.8)	44005 (48.0)	16280 (48.3)	26350 (47.8)	14565 (48.4)	37440 (49.0)	13170 (48.6)	34205 (48.4)	1168685 (48.3)
**Male**	900415 (51.7)	76995 (51.6)	15685 (51.3)	25070 (52.0)	33495 (52.2)	47695 (52.0)	17405 (51.7)	28790 (52.2)	15545 (51.6)	38920 (51.0)	13915 (51.4)	36495 (51.6)	1250415 (51.7)
**Region**													
**North East**	113480 (6.5)	1015 (0.7)	325 (1.1)	1005 (2.1)	840 (1.3)	1290 (1.4)	970 (2.9)	1020 (1.8)	35 (0.1)	595 (0.8)	120 (0.5)	1550 (2.2)	122245 (5.1)
**North West**	258770 (14.9)	7660 (5.1)	2245 (7.3)	3355 (7.0)	4455 (6.9)	13975 (15.2)	3580 (10.6)	7740 (14.0)	985 (3.3)	4465 (5.9)	1280 (4.7)	3790 (5.4)	312305 (12.9)
**Yorkshire and Humber**	202020 (11.6)	4910 (3.3)	1930 (9.0)	3915 (8.1)	3700 (5.8)	20410 (22.3)	1550 (4.6)	3965 (7.2)	830 (2.8)	2745 (3.6)	780 (2.9)	3890 (5.5)	251450 (10.4)
**East Midlands**	152970 (8.8)	3745 (2.5)	4335 (6.3)	3425 (7.1)	6130 (9.6)	2995 (3.3)	810 (2.4)	2430 (4.4)	1115 (3.7)	2340 (3.1)	920 (3.4)	12655 (17.9)	191465 (7.9)
**West Midlands**	181160 (10.4)	11475 (7.7)	2735 (14.2)	4930 (10.2)	13595 (21.2)	23190 (25.3)	4905 (14.6)	3790 (6.9)	5695 (18.9)	5040 (6.6)	1515 (5.6)	6575 (9.3)	266195 (11.0)
**East of England**	203130 (11.7)	14885 (10.0)	3575 (11.7)	5515 (11.4)	4085 (6.4)	7365 (8.0)	2675 (7.9)	3185 (5.8)	1160 (3.8)	4470 (5.9)	765 (2.8)	2920 (4.1)	253725 (10.5)
**London**	160190 (9.2)	64880 (43.5)	8970 (29.4)	15680 (32.5)	22850 (35.6)	14735 (16.1)	16875 (50.1)	25690 (46.6)	18645 (61.9)	49780 (65.2)	20255 (74.8)	32260 (45.6)	450810 (18.6)
**South East**	281350 (16.1)	34540 (23.1)	3730 (12.2)	7045 (14.6)	6300 (9.8)	6980 (7.6)	1815 (5.4)	5865 (10.6)	1050 (3.5)	4975 (6.5)	950 (3.5)	5255 (7.4)	359850 (14.9)
**South West**	189110 (10.9)	6195 (4.1)	2695 (8.8)	3310 (6.9)	2170 (3.4)	760 (0.8)	505 (1.5)	1460 (2.6)	590 (2.0)	1950 (2.6)	500 (1.8)	1810 (2.6)	211055 (8.7)
**IMD Quintile of Deprivation**													
**1 (most)**	396765 (22.8)	38565 (25.9)	12005 (39.4)	13860 (28.8)	19625 (30.6)	55040 (60.1)	23695 (70.4)	19245 (35.0)	16420 (54.6)	42130 (55.3)	14710 (54.4)	28270 (40.0)	680330 (28.2)
**2**	355930 (20.5)	34170 (22.9)	7830 (25.7)	11035 (22.9)	17395 (27.2)	20125 (22.0)	5720 (17.0)	14925 (27.1)	8320 (27.7)	20305 (26.6)	7850 (29.0)	17845 (25.3)	521450 (21.6)
**3**	336295 (19.3)	26385 (17.7)	4790 (15.7)	8620 (17.9)	12080 (18.9)	9075 (9.9)	2335 (6.9)	9525 (17.3)	3290 (10.9)	8185 (10.7)	2710 (10.0)	11030 (15.6)	434330 (18.0)
**4**	326555 (18.8)	22665 (15.2)	3155 (10.4)	7275 (15.1)	8085 (12.6)	4530 (4.9)	1070 (3.2)	6365 (11.6)	1240 (4.1)	3395 (4.5)	1095 (4.1)	7730 (11.0)	393165 (16.3)
**5 (least)**	324030 (18.6)	27320 (18.3)	2715 (8.9)	7315 (15.2)	6850 (10.7)	2785 (3.0)	825 (2.5)	4975 (9.0)	780 (2.6)	2175 (2.9)	665 (2.5)	5720 (8.1)	386150 (16.0)

Supplementary Table 2 – Characteristics of young people with emergency hospital admissions of any type 2014-2020. *Note all numbers rounded to nearest 5.*

n (%)	White British	White Other	Mixed White-Black	Mixed Other	Indian	Pakistani	Bangladeshi	Asian Other	Black Caribbean	Black African	Black Other	Other	All
**Total**	161480 (76.5)	7145 (3.4)	2825 (1.3)	3910 (1.9)	4360 (2.1)	9500 (4.5)	2815 (1.3)	4305 (2.0)	2220 (1.1)	4855 (2.3)	2335 (1.1)	5220 (2.5)	210975 (100.0)
**Female**	75940 (47.0)	3240 (45.3)	1335 (47.3)	1755 (44.9)	1830 (42.0)	4125 (43.4)	1230 (43.7)	1820 (42.2)	1030 (46.3)	2160 (44.5)	1100 (47.0)	2300 (44.1)	97860 (46.4)
**Male**	85540 (53.0)	3905 (54.7)	1490 (52.7)	2155 (55.1)	2530 (58.0)	5375 (56.6)	1585 (56.3)	2490 (57.8)	1195 (53.7)	2695 (55.5)	1235 (53.0)	2915 (55.9)	113115 (53.6)
**Region**													
**North East**	12320 (7.6)	65 (0.9)	45 (1.5)	90 (2.3)	65 (1.5)	180 (1.9)	80 (2.8)	75 (1.7)	X (X)	X (X)	X (X)	125 (2.4)	13100 (6.2)
**North West**	28355 (17.6)	395 (5.5)	270 (9.5)	355 (9.1)	400 (9.2)	1750 (18.4)	350 (12.5)	560 (13.0)	100 (4.5)	355 (7.3)	155 (6.6)	380 (7.3)	33425 (15.8)
**Yorkshire and Humber**	18690 (11.6)	300 (4.2)	195 (6.9)	310 (7.9)	235 (5.4)	2270 (23.9)	130 (4.6)	300 (6.9)	65 (2.8)	170 (3.5)	80 (3.4)	255 (4.9)	22995 (10.9)
**East Midlands**	13685 (8.5)	255 (3.6)	215 (7.7)	250 (6.4)	560 (12.9)	255 (2.7)	60 (2.1)	205 (4.8)	85 (3.9)	155 (3.2)	90 (3.9)	390 (7.5)	16210 (7.7)
**West Midlands**	17170 (10.6)	520 (7.3)	535 (18.9)	610 (15.5)	1050 (24.0)	2655 (28.0)	440 (15.6)	435 (10.1)	500 (22.6)	470 (9.7)	155 (6.7)	505 (9.7)	25050 (11.9)
**East of England**	17365 (10.8)	790 (11.1)	325 (11.5)	460 (11.8)	265 (6.1)	715 (7.5)	210 (7.5)	250 (5.8)	115 (5.1)	320 (6.6)	125 (5.4)	285 (5.5)	21220 (10.1)
**London**	8820 (5.5)	2755 (38.6)	665 (23.5)	990 (25.3)	1140 (26.1)	885 (9.3)	1365 (48.5)	1935 (45.0)	1205 (54.3)	2760 (56.9)	1500 (64.2)	2540 (48.7)	26560 (12.6)
**South East**	25900 (16.0)	1680 (23.5)	315 (11.1)	590 (15.1)	510 (11.7)	715 (7.5)	140 (4.9)	465 (10.8)	85 (3.9)	435 (9.0)	180 (7.7)	610 (11.7)	31625 (15.0)
**South West**	19175 (11.9)	380 (5.3)	265 (9.3)	260 (6.7)	130 (2.9)	80 (0.8)	45 (1.6)	80 (1.9)	60 (2.6)	150 (3.1)	40 (1.7)	130 (2.5)	20785 (9.9)
**IMD Quintile of Deprivation**													
**1 (most)**	35775 (22.2)	1695 (23.7)	1060 (37.6)	1170 (30.0)	1150 (26.4)	5635 (59.3)	1845 (65.5)	1420 (33.0)	1145 (51.6)	2535 (52.2)	1070 (45.8)	1945 (37.2)	56450 (26.8)
**2**	30900 (19.1)	1595 (22.4)	700 (24.8)	895 (22.8)	1125 (25.8	2070 (21.8)	515 (18.3)	1140 (26.5)	640 (28.9)	1310 (27.0)	730 (31.3)	1330 (25.5)	42950 (20.4)
**3**	31220 (19.3)	1235 (17.3)	450 (15.9)	680 (17.4)	830 (19.1)	955 (10.1)	280 (9.9)	795 (18.5)	265 (11.8)	530 (10.9)	280 (12.0)	800 (15.3)	38315 (18.2)
**4**	31470 (19.5)	1135 (15.9)	345 (12.2)	560 (14.3)	600 (13.8)	490 (5.2)	105 (3.7)	495 (11.5)	95 (4.3)	270 (5.5)	150 (6.3)	565 (10.8)	36275 (17.2)
**5 (least)**	32120 (19.9)	1480 (20.7)	270 (9.5)	605 (15.5)	655 (15.0)	350 (3.7)	75 (2.6)	450 (10.4)	75 (3.4)	215 (4.4)	110 (4.6)	585 (11.2)	36985 (17.5)

Supplementary Table 3 – Characteristics of young people with emergency hospital admissions for stress-related presentations (SPRs) 2014-2020. *Note all numbers rounded to nearest 5.*

n (%)													
	White British	White Other	Mixed White-Black	Mixed Other	Indian	Pakistani	Bangladeshi	Asian Other	Black Caribbean	Black African	Black Other	Other	All
**Total**	55935 (79.0)	2250 (3.2)	945 (1.3)	1290 (1.8)	1235 (1.7)	2920 (4.1)	915 (1.3)	1200 (1.7)	680 (1.0)	1300 (1.8)	685 (1.0)	1480 (2.1)	70845 (100.0)
**Female**	34585 (61.8)	1345 (59.8)	600 (63.5)	760 (58.9)	640 (51.8)	1560 (53.4)	495 (54.1)	645 (53.8)	405 (59.6)	720 (55.4)	420 (61.3)	865 (58.5)	43045 (60.8)
**Male**	21350 (38.2)	900 (40.0)	345 (36.5)	535 (41.5)	595 (48.2)	1360 (46.6)	415 (45.4)	555 (46.3)	275 (40.4)	580 (44.6)	265 (38.7)	615 (41.6)	27795 (39.2)
**Region**													
**North East**	3810 (6.8)	20 (0.9)	10 (1.1)	20 (1.6)	15 (1.2)	40 (1.4)	25 (2.7)	20 (1.7)	X (X)	X (X)	X (X)	35 (2.4)	4015 (5.7)
**North West**	10340 (18.5)	130 (5.8)	100 (10.6)	115 (8.9)	105 (8.5)	495 (17.0)	105 (11.5)	160 (13.3)	35 (5.2)	100 (7.7)	50 (7.3)	110 (7.4)	11855 (16.7)
**Yorkshire and Humber**	5985 (10.7)	95 (4.2)	70 (7.4)	105 (8.1)	70 (5.6)	665 (22.8)	35 (3.8)	75 (6.3)	15 (2.2)	30 (2.3)	15 (2.2)	70 (4.7)	7230 (10.2)
**East Midlands**	4720 (8.4)	85 (3.8)	75 (7.9)	85 (6.6)	135 (10.9)	70 (2.4)	15 (1.6)	55 (4.6)	30 (4.4)	40 (3.1)	30 (4.4)	110 (7.4)	5450 (7.7)
**West Midlands**	6475 (11.6)	165 (7.3)	175 (18.5)	210 (16.3)	326 (26.4)	880 (30.1)	140 (15.3)	130 (10.8)	150 (22.1)	135 (10.4)	45 (6.6)	150 (10.1)	8985 (12.7)
**East of England**	5920 (10.6)	260 (11.6)	100 (10.6)	170 (13.2)	80 (6.4)	230 (7.9)	75 (8.2)	75 (6.3)	30 (4.4)	90 (6.9)	40 (5.8)	90 (6.1)	7155 (10.1)
**London**	2770 (5.0)	840 (37.3)	195 (20.6)	305 (23.6)	325 (26.3)	270 (9.3)	465 (50.8)	530 (44.2)	370 (54.4)	710 (54.6)	430 (62.8)	705 (47.6)	7920 (11.2)
**South East**	8715 (15.6)	515 (22.9)	115 (12.2)	180 (14.0)	145 (11.7)	230 (7.9)	40 (4.4)	130 (10.8)	30 (4.4)	140 (10.8)	55 (8.0)	170 (11.5)	10460 (14.8)
**South West**	7195 (12.9)	135 (6.0)	110 (11.6)	95 (7.4)	40 (3.2)	35 (1.2)	15 (1.6)	30 (2.5)	20 (2.9)	45 (3.5)	10 (1.5)	35 (2.4)	7770 (11.0)
**IMD Quintile of Deprivation**													
**1(most)**	12600 (22.5)	520 (23.1)	350 (37.0)	375 (29.1)	350 (28.3)	1720 (58.9)	625 (68.3)	400 (33.3)	365 (53.7)	660 (50.8)	300 (43.8)	580 (39.2)	18850 (26.6)
**2**	11000 (19.7)	495 (22.0)	245 (25.9)	295 (22.9)	315 (25.5)	655 (22.4)	155 (16.9)	290 (24.2)	185 (27.2)	360 (27.7)	210 (30.7)	335 (22.6)	14540 (20.5)
**3**	11050 (19.8)	395 (17.6)	145 (15.3)	235 (18.2)	220 (17.8)	295 (10.1)	90 (9.8)	230 (19.2)	80 (11.8)	150 (11.5)	85 (12.4)	240 (16.2)	13215 (18.7)
**4**	10735 (19.2)	375 (16.7)	115 (12.2)	185 (14.3)	170 (13.8)	150 (5.1)	25 (2.7)	140 (11.7)	30 (4.4)	75 (5.8)	55 (8.0)	160 (10.8)	12215 (17.2)
**5(least)**	10550 (18.9)	460 (20.4)	90 (9.5)	200 (15.5)	185 (15.0)	100 (3.4)	20 (2.2)	140 (11.7)	20 (2.9)	55 (4.2)	35 (5.1)	165 (11.2)	12025 (17.0)

Supplementary Table 4 – Proportion of all emergency hospital admissions deemed stress-related presentations (SPRs) 2014-2020, stratified by gender

n (%)													
	White British	White Other	Mixed White-Black	Mixed Other	Indian	Pakistani	Bangladeshi	Asian Other	Black Caribbean	Black African	Black Other	Other	All
**Total**	77156 (32.5)	2898 (29.9)	1307 (30.2)	1753 (30.0)	1582 (25.2)	3838 (24.0)	1157 (27.5)	1502 (23.8)	887 (24.9)	1624 (20.6)	891 (25.7)	1889 (26.1)	96484 (30.9)
**Female**	51410 (42.4)	1842 (39.6)	885 (40.0)	1118 (39.7)	867 (31.9)	2146 (29.3)	660 (33.6)	862 (31.2)	559 (31.1)	940 (25.7)	578 (33.3)	1150 (34.2)	63017 (40.3)
**Male**	25746 (22.1)	1056 (20.9)	422 (20.0)	635 (21.0)	715 (20.0)	1692 (19.5)	497 (22.1)	640 (18.0)	328 (18.6)	684 (16.1)	313 (18.1)	739 (19.2)	33467 (21.4)

Supplementary Table 5 – Rates of admissions for stress-related presentations (SPRs) for young people aged 11 to 15 years 2014-2020 per 1,000 births, stratified by gender

Rate per 1,000 (95% CI)													
	White British	White Other	Mixed White-Black	Mixed Other	Indian	Pakistani	Bangladeshi	Asian Other	Black Caribbean	Black African	Black Other	Other	All
**11**	8.23 (8.10 - 8.37)	4.06 (3.74 - 4.38)	8.90 (7.85 - 9.96)	6.95 (6.21 - 7.70)	5.79 (5.20 - 6.37)	10.95 (10.28 - 11.62)	7.57 (6.64 - 8.50)	6.69 (6.01 - 7.37)	6.44 (5.54 - 7.35)	5.33 (4.81 -5.85)	7.20 (6.19 - 8.21)	5.53 (4.98 - 6.08)	7.75 (7.64 -7.86)
**Female**	8.67 (8.47 - 8.86)	4.12 (3.65 - 4.59)	8.95 (7.43 - 10.46)	6.62 (5.57 - 7.67)	5.78 (4.93 - 6.63)	11.14 (10.16 - 12.12)	6.88 (5.61 - 8.15)	6.34 (5.38 - 7.30)	6.66 (5.34 - 7.98)	5.37 (4.63 - 6.11)	7.75 (6.25 - 9.24)	5.17 (4.41 - 5.94)	8.04 (7.88 - 8.21)
**Male**	7.83 (7.65 - 8.01)	4.00 (3.55 - 4.45)	8.86 (7.40 - 10.33)	7.26 (6.21 - 8.31)	5.79 (4.98 - 6.60)	10.78 (9.85 - 11.70)	8.22 (6.88 - 9.56)	7.02 (6.05 - 7.98)	6.24 (5.00 - 7.48)	5.29 (4.57 - 6.01)	6.68 (5.33 - 8.04)	5.86 (5.08 - 6.65)	7.47 (7.32 - 7.62)
**12**	11.59 (11.41 - 11.76)	5.84 (5.40 - 6.29)	11.86 (10.48 - 13.25)	10.36 (9.32 - 11.39)	7.77 (6.99 - 8.55)	13.33 (12.49 - 14.18)	8.94 (7.80 - 10.07)	8.47 (7.60 - 9.34)	7.66 (6.57 - 8.76)	7.44 (6.74 - 8.14)	9.75 (8.45 - 11.05)	8.66 (7.87 - 9.45)	10.81 (10.67 - 10.96)
**Female**	14.15 (13.87 - 14.43)	7.19 (6.48 - 7.91)	15.19 (12.95 - 17.43)	12.44 (10.81 - 14.08)	7.93 (6.79 - 9.07)	13.91 (12.67 - 15.16)	9.48 (7.80 - 11.16)	9.74 (8.39 - 11.10)	9.76 (7.98 - 11.54)	8.12 (7.08 - 9.16)	11.68 (9.65 - 13.72)	9.83 (8.62 - 11.04)	13.01 (12.77 - 13.24)
**Male**	9.19 (8.97 - 9.41)	4.58 (4.03 - 5.14)	8.68 (7.02 - 10.34)	8.43 (7.14 - 9.73)	7.62 (6.56 - 8.69)	12.81 (11.66 - 13.95)	8.43 (6.89 - 9.97)	7.30 (6.18 - 8.42)	5.72 (4.40 - 7.03)	6.79 (5.85 - 7.72)	7.92 (6.28 - 9.55)	7.56 (6.54 - 8.59)	8.76 (8.58 - 8.95)
**13**	16.36 (16.11 - 16.60)	8.17 (7.52 - 8.81)	17.57 (15.58 - 19.57)	15.24 (13.74 - 16.74)	9.75 (8.72 - 10.77)	15.40 (14.33 - 16.46)	12.48 (10.90 - 14.05)	10.39 (9.24 - 11.54)	10.59 (9.09 - 12.09)	8.89 (7.97 - 9.81)	10.41 (8.86 - 11.97)	11.14 (10.06 - 12.23)	15.03 (14.83 - 15.23)
**Female**	23.79 (23.37 - 24.21)	10.92 (9.85 - 11.98)	27.82 (24.25 - 31.38)	22.41 (19.79 - 25.02)	11.28 (9.68 - 12.88)	18.45 (16.76 - 20.13)	15.94 (13.38 - 18.50)	13.22 (11.34 - 15.09)	15.23 (12.64 - 17.82)	11.53 (10.03 - 13.03)	15.19 (12.50 - 17.87)	15.64 (13.79 - 17.50)	21.50 (21.16 - 21.85)
**Male**	9.39 (9.13 - 9.65)	5.59 (4.85 - 6.33)	7.75 (5.89 - 9.62)	8.62 (7.05 - 10.19)	8.35 (7.04 - 9.66)	12.63 (11.30 - 13.97)	9.23 (7.35 - 11.12)	7.83 (6.45 - 9.21)	6.32 (4.71 - 7.92)	6.37 (5.28 - 7.46)	5.92 (4.28 - 7.55)	6.97 (5.77 - 8.17)	8.98 (8.76 - 9.20)
**14**	25.74 (25.36 - 26.12)	12.76 (11.74 - 13.78)	24.30 (21.37 - 27.24)	23.60 (21.24 - 25.96)	12.53 (11.07 - 13.99)	20.69 (19.15 - 22.23)	20.40 (17.89 - 22.91)	14.01 (12.31 - 15.70)	14.04 (11.93 - 16.15)	12.14 (10.73 - 13.54)	16.95 (14.52 - 19.37)	17.42 (15.68 - 19.16)	23.38 (23.06 - 23.69)
**Female**	40.61 (39.93 - 41.29)	19.85 (18.03 - 21.67)	36.97 (31.83 - 42.12)	35.99 (31.81 - 40.18)	17.86 (15.34 - 20.38)	28.74 (26.11 - 31.36)	27.53 (23.37 - 31.70)	19.62 (16.72 - 22.52)	18.79 (15.26 - 22.32)	16.13 (13.82 - 18.45)	24.57 (20.38 - 28.76)	25.77 (22.73 - 28.81)	36.35 (35.79 - 36.90)
**Male**	11.83 (11.47 - 12.19)	6.10 (5.12 - 7.09)	12.20 (9.28 - 15.13)	12.26 (9.90 - 14.63)	7.69 (6.10 - 9.27)	13.43 (11.71 - 15.15)	13.68 (10.81 - 16.56)	8.95 (7.07 - 10.82)	9.68 (7.24 - 12.11)	8.31 (6.68 - 9.94)	9.81 (7.23 - 12.39)	9.64 (7.83 - 11.45)	11.27 (10.97 - 11.57)
**15**	38.38 (37.72 - 39.03)	19.43 (17.63 - 21.24)	41.76 (36.19 - 47.33)	37.60 (33.21 - 41.99)	19.12 (16.54 - 21.71)	26.06 (23.59 - 28.54)	29.09 (24.91 - 33.28)	23.81 (20.57 - 27.05)	25.28 (21.29 - 29.28)	13.73 (11.53 - 15.93)	23.01 (19.06 - 26.97)	25.09 (22.11 - 28.07)	34.79 (34.25 - 35.33)
**Female**	60.40 (59.22 - 61.57)	28.71 (25.58 - 31.84)	67.51 (57.41 - 77.61)	57.89 (50.02 - 65.75)	24.42 (20.19 - 28.65)	37.64 (33.35 - 41.93)	38.55 (31.70 - 45.40)	34.19 (28.56 - 39.82)	37.88 (30.94 - 44.82)	18.85 (15.17 - 22.53)	37.60 (30.41 - 44.80)	35.59 (30.55 - 40.63)	54.04 (53.08 - 55.01)

**Male**	17.79 (17.16 - 18.42)	10.61 (8.74 - 12.48)	18.17 (13.02 - 23.31)	19.63 (15.23 - 24.03)	14.33 (11.23 - 17.43)	15.62 (12.96 - 18.27)	19.99 (15.10 - 24.87)	14.61 (11.11 - 18.10)	13.21 (9.14 - 17.27)	8.80 (6.32 - 11.28)	9.18 (5.67 - 12.69)	14.99 (11.75 - 18.23)	16.81 (16.27 - 17.34)

Supplementary Figure 2 – Yearly incidence of emergency hospital admissions for stress-related presentations (SPRs) 2014-2020 per 1,000 births of young people born 2003-2004

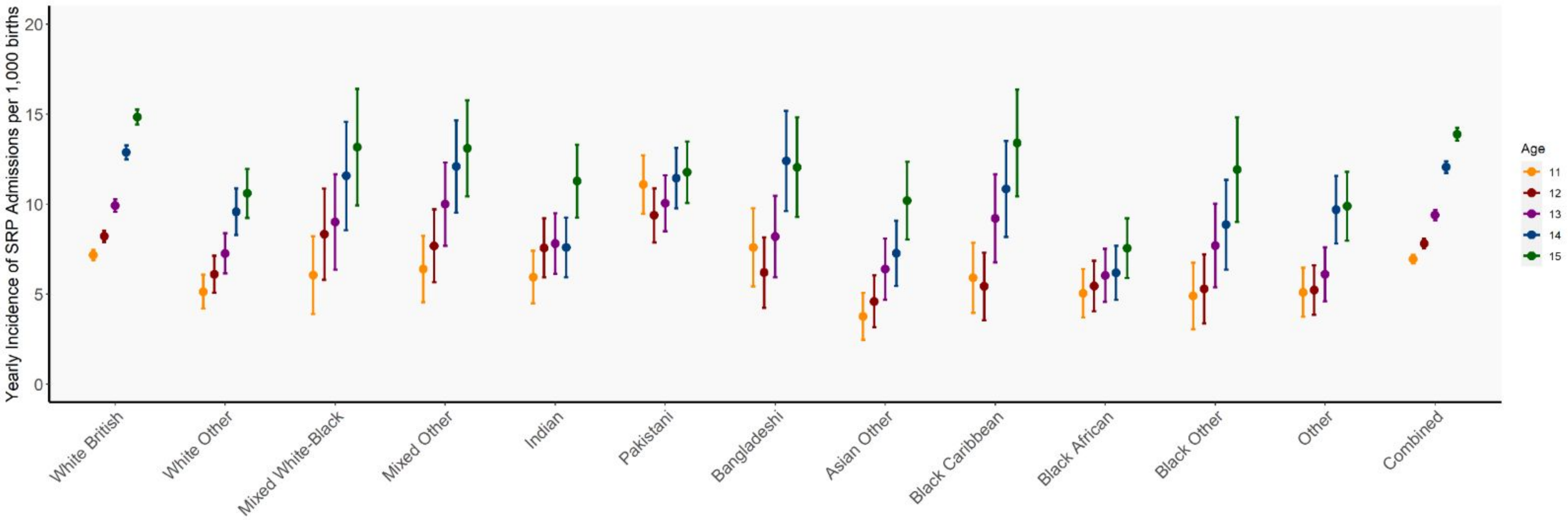

Supplementary Table 6 – Cumulative Incidence between ages 11 and 15 of emergency hospital admissions for stress-related presentations (SPRs) 2014-2020 of young people born 2003-2004, stratified by gender

% (95% CI)	White British	White Other	Mixed White-Black	Mixed Other	Indian	Pakistani	Bangladeshi	Asian Other	Black Caribbean	Black African	Black Other	Other	All
**11**	0.72 (0.69-0.75)	0.51 (0.42-0.61)	0.61 (0.39-0.82)	0.64 (0.45-0.82)	0.59 (0.45-0.74)	1.11 (0.95-1.27)	0.76 (0.54-0.98)	0.38 (0.25-0.51)	0.59 (0.40-0.78)	0.50 (0.37-0.64)	0.49 (0.31-0.67)	0.51 (0.37-0.64)	0.69 (0.67-0.72)
**Female**	0.74 (0.69-0.78)	0.56 (0.42-0.70)	0.76 (0.41-1.11)	0.71 (0.43-0.99)	0.55 (0.34-0.75)	1.06 (0.83-1.29)	0.59 (0.32-0.87)	0.37 (0.19-0.56)	0.55 (0.28-0.82)	0.59 (0.38-0.80)	0.56 (0.28-0.84)	0.52 (0.32-0.71)	0.71 (0.68-0.75)
**Male**	0.70 (0.66-0.74)	0.47 (0.34-0.59)	0.46 (0.20-0.73)	0.58 (0.34-0.82)	0.64 (0.43-0.84)	1.16 (0.93-1.39)	0.92 (0.59-1.25)	0.38 (0.20-0.55)	0.63 (0.35-0.91)	0.42 (0.25-0.59)	0.42 (0.18-0.66)	0.50 (0.31-0.69)	0.68 (0.64-0.71)
**12**	1.54 (1.50-1.58)	1.12 (0.98-1.26)	1.44 (1.10-1.77)	1.41 (1.13-1.68)	1.35 (1.13-1.57)	2.05 (1.83-2.27)	1.38 (1.09-1.67)	0.83 (0.64-1.03)	1.13 (0.86-1.40)	1.05 (0.86-1.24)	1.02 (0.75-1.28)	1.03 (0.84-1.22)	1.47 (1.44-1.51)
**Female**	1.70 (1.64-1.76)	1.34 (1.12-1.56)	1.95 (1.39-2.51)	1.63 (1.20-2.06)	1.25 (0.95-1.56)	2.12 (1.80-2.45)	1.22 (0.83-1.62)	0.90 (0.61-1.19)	1.24 (0.84-1.65)	1.17 (0.87-1.46)	1.23 (0.81-1.65)	1.20 (0.90-1.49)	1.63 (1.57-1.68)
**Male**	1.39 (1.33-1.44)	0.92 (0.74-1.09)	0.97 (0.59-1.35)	1.21 (0.86-1.55)	1.44 (1.13-1.75)	1.98 (1.68-2.28)	1.53 (1.10-1.96)	0.78 (0.52-1.03)	1.03 (0.67-1.39)	0.94 (0.68-1.19)	0.81 (0.48-1.14)	0.87 (0.62-1.12)	1.33 (1.29-1.38)
**13**	2.53 (2.47-2.58)	1.85 (1.67-2.02)	2.34 (1.91-2.76)	2.41 (2.05-2.76)	2.13 (1.86-2.41)	3.05 (2.78-3.32)	2.20 (1.83-2.57)	1.47 (1.22-1.73)	2.05 (1.69-2.42)	1.65 (1.41-1.90)	1.79 (1.43-2.14)	1.64 (1.40-1.88)	2.41 (2.37-2.46)
**Female**	3.09 (3.00-3.17)	2.24 (1.96-2.52)	3.15 (2.44-3.87)	3.10 (2.51-3.69)	2.05 (1.66-2.44)	3.20 (2.80-3.60)	2.22 (1.70-2.75)	1.76 (1.35-2.17)	2.46 (1.90-3.03)	1.96 (1.58-2.33)	2.21 (1.65-2.77)	1.94 (1.56-2.31)	2.91 (2.84-2.98)
**Male**	2.01 (1.94-2.08)	1.48 (1.26-1.70)	1.59 (1.11-2.08)	1.79 (1.37-2.21)	2.21 (1.82-2.60)	2.91 (2.55-3.28)	2.17 (1.66-2.69)	1.22 (0.90-1.54)	1.66 (1.20-2.12)	1.36 (1.05-1.67)	1.38 (0.95-1.81)	1.36 (1.05-1.67)	1.95 (1.90-2.01)
**14**	3.82 (3.75-3.88)	2.81 (2.59-3.02)	3.49 (2.98-4.01)	3.61 (3.18-4.05)	2.89 (2.57-3.21)	4.19 (3.88-4.51)	3.44 (2.98-3.90)	2.20 (1.89-2.51)	3.14 (2.69-3.59)	2.27 (1.99-2.55)	2.67 (2.24-3.10)	2.61 (2.30-2.92)	3.62 (3.56-3.68)
**Female**	4.99 (4.88-5.10)	3.63 (3.28-3.99)	4.94 (4.05-5.83)	4.69 (3.96-5.41)	2.96 (2.49-3.43)	4.65 (4.17-5.13)	3.71 (3.03-4.39)	2.54 (.05-3.04)	3.81 (3.10-4.51)	2.73 (2.29-3.18)	3.43 (2.73-4.13)	3.33 (2.84-3.82)	4.65 (4.56-4.74)
**Male**	2.73 (2.65-2.81)	2.02 (1.76-2.28)	2.18 (1.61-2.75)	2.67 (2.15-3.19)	2.82 (2.39-3.26)	3.78 (3.37-4.20)	3.18 (2.56-3.80)	1.89 (1.49-2.29)	2.50 (1.94-3.06)	1.83 (1.47-2.18)	1.96 (1.44-2.47)	1.92 (1.55-2.29)	2.66 (2.59-2.73)
**15**	5.30 (5.22-5.38)	3.86 (3.61-4.12)	4.81 (4.20-5.42)	4.92 (4.41-5.43)	4.02 (3.64-4.39)	5.37 (5.01-5.73)	4.64 (4.11-5.18)	3.22 (2.84-3.60)	4.48 (3.94-5.01)	3.02 (2.70-3.35)	3.86 (3.35-4.38)	3.60 (3.24-3.96)	5.01 (4.94-5.07)
**Female**	7.13 (7.00-7.26)	4.98 (4.56-5.39)	6.58 (5.56-7.60)	6.64 (5.78-7.50)	4.29 (3.73-4.85)	6.32 (5.76-6.89)	5.28 (4.47-6.09)	3.85 (3.25-4.45)	5.84 (4.97-6.71)	3.73 (3.21-4.25)	5.05 (4.21-5.89)	4.86 (4.27-5.46)	6.63 (6.52-6.74)
**Male**	3.61 (3.52-3.70)	2.81 (2.51-3.12)	3.21 (2.52-3.90)	3.42 (2.84-4.01)	3.77 (3.27-4.27)	4.51 (4.06-4.97)	4.03 (3.33-4.73)	2.66 (2.19-3.13)	3.18 (2.54-3.81)	2.35 (1.94-2.76)	2.75 (2.14-3.36)	2.39 (1.98-2.80)	3.51 (3.43-3.58)

Supplementary Table 7 - Trends in the proportion of young people with singular and repeat SRP admissions across racial-ethnic groups, stratified by gender

% (95% CI)													
	White British	White Other	Mixed White-Black	Mixed Other	Indian	Pakistani	Bangladeshi	Asian Other	Black Caribbean	Black African	Black Other	Other	All
**1**	58.6 (58.2-59.0)	64.2 (62.2-66.1)	58.7 (55.6-61.8)	60.5 (57.9-63.2)	60.9 (58.2-63.6)	52.3 (50.5-54.1)	61.8 (58.7-65.0)	60.2 (57.4-63.0)	59.3 (55.6-63.0)	60.1 (57.4-62.8)	63.6 (60.0-67.3)	62.0 (59.5-64.5)	58.8 (58.5-59.2)
**Female**	55.8 (55.3-56.3)	61.6 (59.0-64.2)	55.7 (51.7-59.6)	56.6 (53.1-60.1)	60.9 (57.2-64.7)	52.6 (50.1-55.1)	61.8 (57.5-66.0)	60.2 (56.5-64.0)	57.7 (52.9-62.5)	59.8 (56.2-63.4)	61.4 (56.8-66.1)	62.1 (58.8-65.3)	56.4 (55.9-56.8)
**Male**	63.1 (62.5-63.8)	68.0 (64.9-71.0)	64.0 (58.9-69.0)	66.1 (62.1-70.1)	60.8 (56.9-64.7)	51.9 (49.3-54.6)	61.9 (57.2-66.5)	60.1 (56.1-64.2)	61.7 (55.9-67.4)	60.5 (56.5-64.5)	67.2 (61.5-72.8)	62.0 (58.1-65.8)	62.6 (62.0-63.2)
**2**	21.0 (20.6-21.3)	19.8 (18.1-21.4)	19.9 (17.3-22.4)	18.3 (16.2-20.5)	21.3 (19.0-23.5)	22.0 (20.5-23.5)	17.8 (15.4-20.3)	22.1 (19.7-24.4)	19.7 (16.7-22.7)	17.7 (15.6-19.8)	16.9 (14.1-19.7)	21.4 (19.3-23.5)	20.8 (20.5-21.1)
**Female**	21.6 (21.2-22.0)	20.2 (18.0-22.3)	22.3 (19.0-25.7)	19.4 (16.6-22.2)	20.2 (17.0-23.3)	21.1 (19.1-23.2)	17.3 (14.0-20.6)	21.9 (18.7-25.1)	19.9 (16.0-23.8)	18.7 (15.9-21.6)	17.4 (13.8-21.0)	20.8 (18.1-23.5)	21.3 (20.9-21.7)
**Male**	19.9 (19.3-20.4)	19.2 (16.6-21.7)	15.6 (11.7-19.4)	16.9 (13.7-20.0)	22.4 (19.1-25.8)	22.9 (20.7-25.1)	18.5 (14.7-22.2)	22.3 (18.8-25.7)	19.3 (14.7-24.0)	16.4 (13.4-19.4)	16.2 (11.8-20.7)	22.3 (19.0-25.6)	19.9 (19.4-20.4)
**3**	8.8 (8.5-9.0)	8.0 (6.9-9.2)	8.6 (6.8-10.3)	9.3 (7.7-10.9)	7.3 (5.8-8.7)	8.9 (7.8-9.9)	8.4 (6.6-10.2)	7.8 (6.3-9.3)	7.8 (5.8-9.8)	8.0 (6.5-9.5)	7.4 (5.5-9.4)	7.8 (6.5-9.2)	8.6 (8.4-8.9)
**Female**	9.3 (9.0-9.7)	9.1 (7.6-10.7)	9.2 (6.9-11.5)	10.9 (8.7-13.2)	7.8 (5.7-9.9)	8.7 (7.3-10.1)	8.0 (5.7-10.4)	7.5 (5.4-9.5)	9.3 (6.5-12.2)	8.7 (6.7-10.8)	9.0 (6.3-11.8)	8.1 (6.3-9.9)	9.2 (9.0-9.5)
**Male**	7.8 (7.4-8.2)	6.4 (4.8-8.0)	7.5 (4.7-10.3)	6.9 (4.8-9.1)	6.7 (4.7-8.7)	9.1 (7.5-10.6)	8.9 (6.1-11.6)	8.3 (6.0-10.5)	5.5 (2.8-8.2)	7.1 (5.0-9.2)	4.9 (2.3-7.5)	7.5 (5.4-9.6)	7.7 (7.4-8.0)
**4+**	11.7 (11.4-12.0)	8.0 (6.9-9.1)	12.9 (10.7-15.0)	11.8 (10.1-13.6)	10.6 (8.9-12.3)	16.9 (15.5-18.2)	11.9 (9.8-14.0)	9.9 (8.2-11.6)	13.2 (10.7-15.8)	14.2 (12.3-16.1)	12.0 (9.5-14.4)	8.8 (7.3-10.2)	11.8 (11.5-12.0)
**Female**	13.2 (12.9-13.6)	9.1 (7.5-10.6)	12.8 (10.2-15.5)	13.1 (10.7-15.5)	11.1 (8.7-13.5)	17.6 (15.7-19.4)	12.9 (9.9-15.8)	10.4 (8.0-12.8)	13.0 (9.8-16.3)	12.8 (10.3-15.2)	12.1 (9.0-15.3)	9.1 (7.2-11.0)	13.1 (12.7-13.4)
**Male**	9.2 (8.8-9.6)	6.4 (4.8-8.0)	13.0 (9.4-16.5)	10.1 (7.6-12.7)	10.1 (7.6-12.5)	16.1 (14.2-18.1)	10.8 (7.8-13.8)	9.3 (6.9-11.8)	13.5 (9.5-17.6)	16.0 (13.0-19.0)	11.7 (7.8-15.6)	8.3 (6.1-10.5)	9.8 (9.4-10.1)

Supplementary Table 8 - Trends in the proportion of SRP admissions with a duration of 0, 1, 2, 3-4, 5+ days across racial-ethnic groups, stratified by gender

% (95% CI)	White British	White Other	Mixed White-Black	Mixed Other	Indian	Pakistani	Bangladeshi	Asian Other	Black Caribbean	Black African	Black Other	Other	All
**0**	45.1 (44.7-45.4)	44.9 (43.1-46.7)	41.2 (38.5-43.8)	43.0 (40.7-45.3)	46.7 (44.3-49.2)	44.6 (43.0-46.2)	41.7 (38.8-44.5)	45.1 (42.6-47.6)	45.3 (42.0-48.6)	47.8 (45.4-50.2)	42.6 (39.4-45.9)	43.4 (41.2-45.6)	44.9 (44.6-45.3)
**Female**	42.0 (41.6-42.4)	42.8 (40.6-45.1)	39.2 (36.0-42.4)	39.5 (36.7-42.4)	43.9 (40.6-47.2)	42.5 (40.4-44.5)	40.3 (36.6-44.0)	42.1 (38.8-45.4)	43.1 (39.0-47.2)	46.5 (43.3-49.7)	41.0 (37.0-45.0)	40.2 (37.3-43.0)	42.0 (41.6-42.4)
**Male**	51.2 (50.6-51.8)	48.5 (45.5-51.5)	45.3 (40.5-50.0)	49.1 (45.2-53.0)	50.1 (46.4-53.7)	47.3 (45.0-49.7)	43.5 (39.1-47.8)	49.1 (45.2-52.9)	49.1 (43.7-54.5)	49.6 (45.8-53.3)	45.7 (40.2-51.2)	48.4 (44.8-52.0)	50.5 (49.9-51.0)
**1**	33.7 (33.4-34.1)	32.6 (30.9-34.4)	34.1 (31.6-36.7)	32.2 (30.0-34.4)	31.0 (28.7-33.3)	32.3 (30.8-33.8)	32.0 (29.3-34.7)	29.4 (27.1-31.7)	30.3 (27.3-33.4)	28.1 (25.9-30.3)	30.4 (27.4-33.4)	31.4 (29.3-33.5)	33.3 (33.0-33.6)
**Female**	34.7 (34.2-35.1)	33.0 (30.9-35.2)	35.3 (32.1-38.4)	31.9 (29.2-34.7)	31.7 (28.6-34.8)	32.6 (30.6-34.6)	31.1 (27.5-34.6)	30.9 (27.8-33.9)	31.7 (27.8-35.5)	28.3 (25.4-31.2)	29.4 (25.7-33.1)	33.0 (30.2-35.7)	34.2 (33.8-34.5)
**Male**	31.9 (31.4-32.5)	32.0 (29.2-34.8)	31.8 (27.3-36.2)	32.6 (29.0-36.2)	30.1 (26.7-33.4)	31.9 (29.6-34.1)	33.2 (29.1-37.3)	27.5 (24.0-31.0)	28.0 (23.2-32.9)	27.8 (24.4-31.1)	32.3 (27.1-37.4)	29.0 (25.7-32.2)	31.7 (31.2-32.2)
**2**	9.2 (9.0-9.4)	9.2 (8.2-10.3)	9.6 (8.0-11.2)	10.0 (8.6-11.4)	10.5 (9.0-12.0)	9.8 (8.8-10.7)	10.1 (8.4-11.8)	9.6 (8.1-11.1)	8.6 (6.7-10.4)	10.0 (8.6-11.5)	10.3 (8.3-12.3)	10.5 (9.1-11.9)	9.4 (9.2-9.5)
**Female**	9.9 (9.7-10.2)	9.8 (8.4-11.1)	9.0 (7.2-10.9)	10.8 (9.0-12.6)	11.4 (9.3-13.5)	10.5 (9.2-11.8)	11.4 (8.9-13.8)	10.1 (8.1-12.1)	8.1 (5.8-10.3)	10.1 (8.2-12.0)	11.8 (9.1-14.4)	11.2 (9.4-13.0)	10.0 (9.8-10.2)
**Male**	7.8 (7.5-8.2)	8.2 (6.6-9.9)	10.7 (7.7-13.6)	8.5 (6.3-10.7)	9.4 (7.2-11.5)	8.8 (7.5-10.2)	8.5 (6.0-10.9)	8.9 (6.7-11.1)	9.5 (6.3-12.6)	9.9 (7.7-12.2)	7.7 (4.7-10.6)	9.3 (7.2-11.4)	8.1 (7.8-8.4)
**3-4**	6.0 (5.9-6.2)	5.9 (5.1-6.8)	7.8 (6.3-9.3)	6.7 (5.6-7.9)	6.7 (5.5-7.9)	7.2 (6.4-8.1)	8.5 (6.9-10.1)	7.7 (6.4-9.1)	6.4 (4.8-8.0)	6.5 (5.3-7.7)	7.9 (6.1-9.6)	6.9 (5.7-8.0)	6.2 (6.1-6.4)
**Female**	6.6 (6.4-6.8)	6.2 (5.1-7.3)	8.6 (6.7-10.4)	8.9 (7.2-10.5)	7.3 (5.5-9.0)	7.3 (6.2-8.4)	9.1 (6.9-11.3)	7.7 (5.9-9.4)	7.3 (5.2-9.5)	7.4 (5.8-9.1)	8.5 (6.2-10.7)	7.5 (6.0-9.0)	6.8 (6.6-7.0)
**Male**	4.9 (4.6-5.1)	5.5 (4.1-6.9)	6.2 (3.9-8.5)	3.0 (1.7-4.3)	6.0 (4.3-7.8)	7.2 (5.9-8.4)	7.6 (5.3-10.0	7.8 (5.7-9.9)	4.9 (2.5-7.2)	5.3 (3.6-6.9)	6.7 (3.9-9.5)	6.0 (4.2-7.7)	5.1 (4.9-5.4)
**5+**	5.9 (5.7-6.1)	7.3 (6.4-8.3)	7.3 (5.9-8.8)	8.1 (6.8-9.4)	5.1 (4.0-6.2)	6.1 (5.3-6.9)	7.8 (6.2-9.3)	8.2 (6.8-9.6)	9.4 (7.4-11.3)	7.6 (6.3-8.9)	8.8 (6.9-10.6)	7.8 (6.6-9.0)	6.2 (6.0-6.3)
**Female**	6.8 (6.6-7.0)	8.2 (6.9-9.5)	7.9 (6.1-9.7)	8.9 (7.2-10.5)	5.7 (4.1-7.2)	7.1 (6.0-8.2)	8.2 (6.1-10.3)	9.3 (7.3-11.2)	9.8 (7.4-12.3)	7.7 (6.0-9.4)	9.3 (7.0-11.7)	8.2 (6.6-9.8)	7.0 (6.8-7.2)
**Male**	4.1 (3.9-4.4)	5.8 (4.4-7.2)	6.2 (3.9-8.5)	6.8 (4.8-8.7)	4.5 (3.0-6.0)	4.8 (3.8-5.9)	7.2 (5.0-9.5)	6.7 (4.8-8.7)	8.5 (5.5-11.6)	7.5 (5.5-9.4)	7.7 (4.7-10.6)	7.3 (5.4-9.2)	4.6 (4.4-4.8)

Supplementary Table 9 – Proportion of SRP Admissions with a recorded diagnoses related to (a) Psychosomatic (b) Internalising (c) Externalising (d) Thought disorder (e) Self-harm symptoms. Note: *Results for Mixed White-Black race/ethnicity, Mixed Other race/ethnicity and Indian groups with recorded diagnoses related to Thought disorder censored due to small cell count, gender breakdown not available due to small cell counts*

% (95% CI)	White British	White Other	Mixed White-Black	Mixed Other	Indian	Pakistani	Bangladeshi	Asian Other	Black Caribbean	Black African	Black Other	Other	All
**Psycho-somatic**	75.9 (75.6-76.2)	78.2 (76.7-79.7)	75.4 (73.1-77.8)	77.4 (75.4-79.3)	89.3 (87.8-90.8)	92.7 (91.9-93.5)	86.9 (84.9-88.8)	87.9 (86.3-89.6)	80.9 (78.4-83.5)	88.6 (87.1-90.2)	80.4 (77.8-83.0)	81.9 (80.2-83.6)	77.6 (77.3-77.9)
**Female**	69.9 (69.5-70.3)	72.3 (70.2-74.3)	71.5 (68.6-74.5)	71.4 (68.7-74.0)	84.3 (81.9-86.7)	89.4 (88.1-90.7)	82.0 (79.0-84.9)	82.8 (80.3-85.3)	75.1 (71.6-78.7)	84.4 (82.0-86.7)	74.4 (70.8-78.0)	76.9 (74.4-79.3)	71.6 (71.3-72.0)
**Male**	87.8 (87.4-88.2)	88.6 (86.7-90.6)	83.6 (80.1-87.2)	87.9 (85.3-90.4)	95.4 (93.8-96.9)	96.8 (96.0-97.6)	93.4 (91.2-95.5)	94.8 (93.1-96.6)	90.9 (87.7-94.0)	94.4 (92.7-96.2)	91.4 (88.3-94.5)	89.7 (87.5-91.9)	88.9 (88.5-89.2)
**Internalising**	15.9 (15.6-16.1)	16.4 (15.1-17.8)	14.8 (12.9-16.8)	16.2 (14.5-17.9)	8.2 (6.8-9.5)	5.9 (5.1-6.6)	8.5 (6.9-10.1)	10.5 (9.0-12.1)	9.5 (7.5-11.4)	7.1 (5.8-8.3)	10.5 (8.5-12.6)	13.4 (11.9-15.0)	14.9 (14.6-15.1)
**Female**	19.0 (18.6-19.3)	20.7 (18.8-22.5)	17.5 (15.0-20.0)	19.8 (17.4-22.1)	10.3 (8.2-12.3)	7.8 (6.6-8.9)	11.5 (9.1-14.0)	13.3 (11.1-15.6)	12.3 (9.6-15.1)	9.0 (7.2-10.9)	11.8 (9.1-14.4)	16.1 (14.0-18.2)	18.0 (17.7-18.3)
**Male**	9.7 (9.3-10.0)	9.0 (7.3-10.7)	9.2 (6.5-12.0)	9.9 (7.6-12.2)	5.6 (3.9-7.3)	3.4 (2.6-4.3)	4.4 (2.6-6.2)	6.7 (4.8-8.7)	4.6 (2.3-6.8)	4.4 (2.9-5.9)	8.3 (5.2-11.4)	9.3 (7.2-11.4)	8.9 (8.6-9.2)
**Externalising**	8.5 (8.3-8.7)	6.4 (5.5-7.3)	8.3 (6.8-9.8)	7.1 (5.9-8.3)	2.1 (1.4-2.8)	1.8 (1.4-2.2)	2.4 (1.5-3.3)	3.2 (2.3-4.1)	7.6 (5.8-9.3)	2.3 (1.6-3.1)	4.7 (3.3-6.1)	5.4 (4.4-6.4)	7.7 (7.5-7.8)
**Female**	7.6 (7.4-7.9)	5.1 (4.1-6.1)	7.0 (5.3-8.7)	6.0 (4.6-7.4)	1.7 (0.9-2.6)	1.4 (0.9-2.6)	1.7 (0.7-2.6)	3.2 (2.1-4.4)	6.1 (4.1-8.1)	2.1 (1.2-3.1)	3.1 (1.7-4.5)	5.7 (4.4-7.1)	6.9 (6.7-7.1)
**Male**	10.2 (9.9-10.6)	8.6 (6.9-10.3)	11.1 (8.1-14.1)	9.0 (6.8-11.2)	2.5 (1.4-3.7)	2.4 (1.7-3.2)	3.4 (1.8-5.0)	3.1 (1.8-4.5)	10.1 (6.8-13.3)	2.6 (1.4-3.8)	7.7 (4.7-10.6)	4.9 (3.3-6.4)	9.1 (8.8-9.4)
**Thought Disorders**	0.46 (0.41-0.51)	1.00 (0.64-1.36)	X	X	X	0.39 (0.19-0.59)	0.86 (0.33-1.40)	1.07 (0.55-1.58)	1.69 (0.84-2.54)	1.35 (0.79-1.92)	2.47 (1.45-3.49)	1.11 (0.64-1.58)	0.54 (0.50-0.59
**Self-Harm**	33.5 (33.2-33.9)	29.3 (27.7-31.0)	35.0 (32.4-37.6)	32.9 (30.7-35.1)	15.4 (13.6-17.2)	10.1 (9.2-11.1)	17.7 (15.5-19.9)	18.2 (16.2-20.1)	28.1 (25.1-31.0)	15.3 (13.5-17.0)	27.2 (24.2-30.1)	25.7 (23.7-27.6)	31.2 (30.9-31.5)
**Female**	42.6 (42.2-43.0)	38.4 (36.2-40.6)	41.4 (38.1-44.6)	43.9 (41.0-46.8)	23.3 (20.5-26.1)	15.6 (14.0-17.1)	27.3 (23.9-30.7)	26.7 (23.7-29.6)	36.9 (32.9-40.9)	22.8 (20.1-25.4)	34.9 (31.1-38.8)	34.1 (31.3-36.8)	40.4 (40.0-40.7)
**Male**	15.4 (15.0-15.9)	13.5 (11.5-15.6)	21.6 (17.6-25.5)	13.5 (10.9-16.2)	5.9 (4.2-7.6)	3.2 (2.4-4.0)	5.0 (3.1-7.0)	6.7 (4.8-8.7)	13.1 (9.5-16.8)	5.0 (3.3-6.6)	12.8 (9.1-16.5)	12.6 (10.2-15.0)	40.4 (40.0-40.7)

Supplementary Table 10 – Proportion of recorded SRP-related diagnoses during SRP Admissions across racial-ethnic groups, stratified by gender

% (95% CI)	White British	White Other	Mixed White-Black	Mixed Other	Indian	Pakistani	Bangladeshi	Asian Other	Black Caribbean	Black African	Black Other	Other	All
**1**	74.2 (73.9-74.5)	75.9 (74.4-77.5)	73.8 (71.5-76.2)	73.9 (71.8-75.9)	87.4 (85.8-89.1)	91.3 (90.4-92.2)	86.6 (84.6-88.6)	84.0 (82.1-85.8)	78.5 (75.8-81.2)	87.9 (86.3-89.5)	80.4 (77.8-83.0)	78.6 (76.7-80.4)	75.9 (75.6-76.1)
**Female**	70.4 (70.0-70.8)	71.5 (69.4-73.6)	70.8 (67.9-73.8)	68.7 (66.0-71.4)	83.9 (81.4-86.3)	88.5 (87.1-89.8)	80.9 (77.9-83.9)	79.2 (76.5-81.9)	75.0 (71.4-78.5)	84.4 (82.0-86.7)	78.9 (75.6-82.2)	73.6 (71.0-76.1)	71.8 (71.5-72.2)
**Male**	81.9 (81.4-82.4)	83.6 (81.4-85.8)	80.1 (76.3-83.9)	83.0 (80.1-85.9)	91.7 (89.7-93.8)	94.9 (93.8-95.9)	94.2 (92.1-96.2)	90.3 (88.0-92.6)	84.5 (80.5-88.4)	92.7 (90.7-94.6)	83.1 (78.9-87.2)	86.3 (83.9-88.8)	83.5 (83.1-83.9)
**2**	18.3 (18.0-18.5)	17.5 (16.1-18.9)	19.2 (17.1-21.3)	18.9 (17.0-20.7)	10.2 (8.7-11.7)	6.7 (6.0-7.5)	10.5 (8.7-12.2)	12.0 (10.3-13.6)	15.9 (13.5-18.3)	9.6 (8.2-11.0)	14.8 (12.5-17.1)	15.7 (14.0-17.3)	17.2 (17.0-17.5)
**Female**	20.7 (20.4-21.1)	20.4 (18.5-22.2)	21.1 (18.4-23.8)	21.9 (19.5-24.3)	12.7 (10.5-14.9)	8.8 (7.6-10.0)	15.0 (12.3-17.7)	14.8 (12.5-17.2)	18.2 (15.0-21.4)	12.0 (9.9-14.1)	16.1 (13.1-19.1)	19.3 (17.0-21.6)	19.9 (19.6-20.2)
**Male**	13.4 (12.9-13.8)	12.5 (10.5-14.5)	15.2 (11.7-18.6)	13.5 (10.9-16.2)	7.1 (5.2-9.0)	4.1 (3.2-5.1)	4.4 (2.6-6.2)	8.1 (6.0-10.2)	11.9 (8.4-15.4)	6.3 (4.5-8.1)	12.5 (8.8-16.1)	10.0 (7.8-12.2)	12.3 (11.9-12.6)
**3+**	7.5 (7.3-7.7)	6.6 (5.7-7.5)	7.0 (5.6-8.3)	7.2 (6.0-8.5)	2.4 (1.6-3.2)	2.0 (1.5-2.4)	2.9 (2.0-3.9)	4.1 (3.1-5.1)	5.6 (4.1-7.2)	2.5 (1.8-3.3)	4.8 (3.4-6.2)	5.8 (4.7-6.8)	6.9 (6.7-7.1)
**Female**	8.9 (8.6-9.1)	8.1 (6.9-9.4)	8.0 (6.2-9.8)	9.4 (7.7-11.1)	3.5 (2.2-4.7)	2.7 (2.0-3.4)	4.1 (2.6-5.6)	5.9 (4.3-7.5)	6.8 (4.7-8.9)	3.6 (2.4-4.8)	5.0 (3.2-6.8)	7.1 (5.6-8.6)	8.3 (8.1-8.5)
**Male**	4.7 (4.5-5.0)	3.9 (2.7-5.0)	4.7 (2.7-6.8)	3.5 (2.0-4.9)	1.1 (0.3-1.9)	1.0 (0.5-1.5)	1.4 (0.4-2.4)	1.6 (0.6-2.5)	3.7 (1.6-5.7)	1.0 (0.3-1.8)	4.5 (2.2-6.8)	3.7 (2.3-5.0)	4.2 (4.0-4.4)

Supplementary Figure 3 – Relative Risk Visual Summary (To be viewed alongside Supplementary Table 11)

- This figure provides a visual summary of the Relative Risk for outcomes (re-admission, duration, types, multiple diagnoses – described below) where cell shading indicates the magnitude of the point estimate. Point estimates and associated 95% CI are outlined in full in Supplementary Table 11. Reference group for each outcome (column): All racial-ethnic groups combined.
- Re-admission: Proportion of young people with repeat SRP admissions between April 2014 and March 2020 across racial-ethnic groups compared to the overall proportion for all groups combined.
- Duration: Proportion of SRP admissions with a duration ≥3 days across racial-ethnic groups compared to the overall proportion for all groups combined.
- Types: Proportion of SRP Admissions with recorded diagnoses related to (1) Psychosomatic (2) Internalising (3) Externalising (4) Self-harm symptoms compared to the overall proportion for all groups combined. These are not mutually exclusive.
- Multiple Diagnoses: Proportion of SRP Admissions with >1 recorded SRP-related diagnoses across racial-ethnic groups compared to the overall proportion for all groups combined.

Note: Relative risk does not account for person-time.

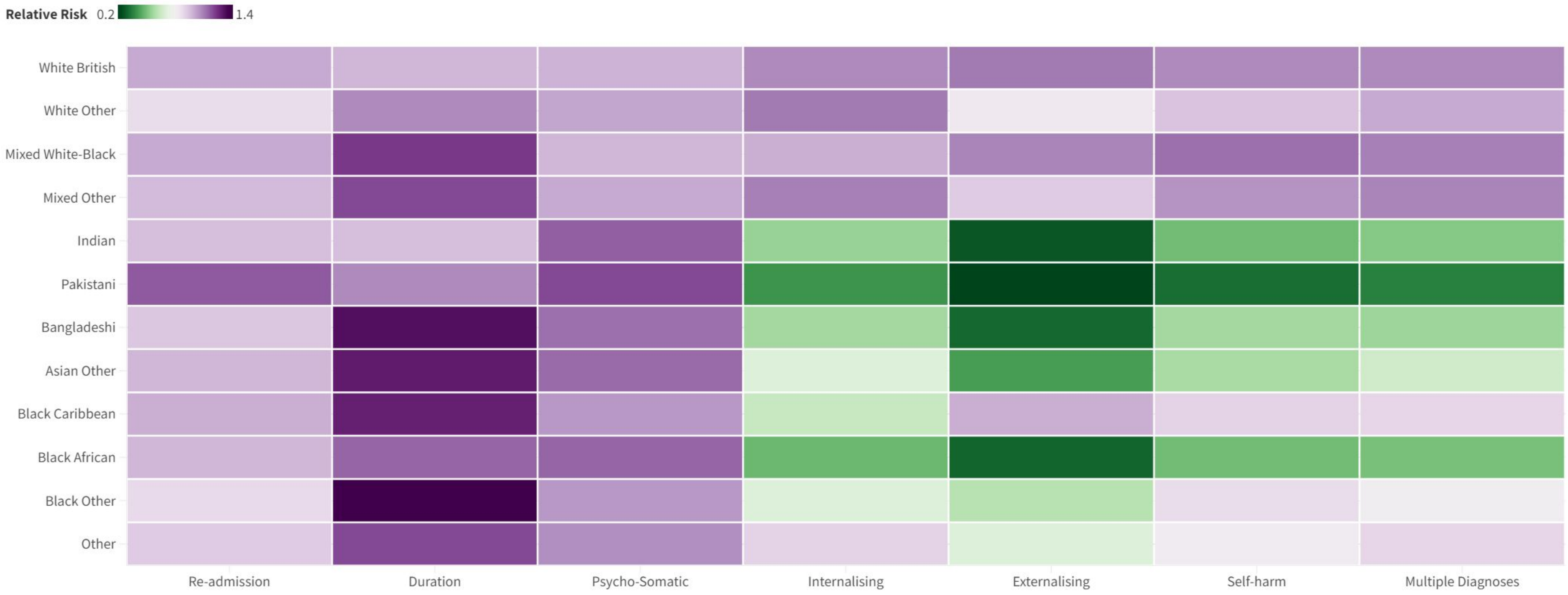

Reference group for each outcome (column): All racial-ethnic groups combined.

Supplementary Table 11 – Relative Risk Data Summary (To accompany Supplementary Figure 3)

Relative Risk (95% CI)							
**White British**	1.00 (0.99-1.02)	0.97 (0.94-0.99)	0.98 (0.97-0.98)	1.07 (1.04-1.09)	1.10 (1.07-1.14)	1.07 (1.06-1.09)	1.07 (1.05-1.09)
**White Other**	0.87 (0.82-0.92)	1.07 (0.98-1.18)	1.01 (0.99-1.03)	1.10 (1.01-1.20)	0.83 (0.72-0.96)	0.94 (0.89-0.99)	1.00 (0.94-1.07)
**Mixed White-Black**	1.00 (0.93-1.08)	1.22 (1.07-1.39)	0.97 (0.94-1.00)	0.99 (0.87-1.13)	1.08 (0.90-1.29)	1.12 (1.04-1.21)	1.09 (0.99-1.19)
**Mixed Other**	0.96 (0.90-1.03)	1.19 (1.07-1.34)	1.00 (0.97-1.02)	1.09 (0.98-1.21)	0.92 (0.78-1.09)	1.05 (0.99-1.13)	1.08 (1.00-1.17)
**Indian**	0.95 (0.88-1.02)	0.95 (0.83-1.09)	1.15 (1.13-1.17)	0.55 (0.47-0.65)	0.27 (0.19-0.38)	0.49 (0.44-0.55)	0.52 (0.46-0.60)
**Pakistani**	1.16 (1.11-1.20)	1.07 (0.99-1.16)	1.19 (1.18-1.21)	0.40 (0.35-0.45)	0.23 (0.19-0.30)	0.32 (0.29-0.36)	0.36 (0.33-0.40)
**Bangladeshi**	0.93 (0.85-1.01)	1.31 (1.15-1.49)	1.12 (1.09-1.15)	0.57 (0.47-0.69)	0.31 (0.22-0.45)	0.57 (0.50-0.64)	0.56 (0.48-0.64)
**Asian Other**	0.97 (0.90-1.04)	1.28 (1.14-1.44)	1.13 (1.11-1.15)	0.70 (0.61-0.82)	0.42 (0.31-0.55)	0.58 (0.52-0.65)	0.66 (0.59-0.75)
**Black Caribbean**	0.99 (0.90-1.08)	1.27 (1.09-1.48)	1.04 (1.01-1.08)	0.64 (0.52-0.78)	0.99 (0.78-1.24)	0.90 (0.81-1.00)	0.89 (0.79-1.01)
**Black African**	0.97 (0.91-1.04)	1.14 (1.01-1.28)	1.14 (1.12-1.16)	0.48 (0.40-0.57)	0.30 (0.22-0.41)	0.49 (0.44-0.55)	0.50 (0.44-0.57)
**Black Other**	0.88 (0.80-0.98)	1.35 (1.16-1.56)	1.04 (1.00-1.07)	0.70 (0.58-0.85)	0.61 (0.45-0.82)	0.87 (0.78-0.97)	0.81 (0.71-0.93)
**Other**	0.92 (0.86-0.98)	1.19 (1.06-1.32)	1.06 (1.03-1.08)	0.90 (0.80-1.01)	0.70 (0.58-0.85)	0.82 (0.74-0.92)	0.89 (0.81-0.97)
	**Re-admission**	**Duration**	**Psycho-Somatic**	**Internalising**	**Externalising**	**Self-harm**	**Multiple Diagnoses**

Reference group for each outcome (column): All racial-ethnic groups combined.

Supplementary Figure 4 – Relative Risk Visual Summary: Thought Disorders (To be viewed alongside Supplementary Table 12)

- Thought Disorders: Proportion of SRP Admissions with a recorded diagnoses related to Thought Disorder symptoms compared to the overall proportion for all groups combined. *Note: Only includes racial-ethnic groups with cell count ≥10.*

Note: Relative risk does not account for person-time.

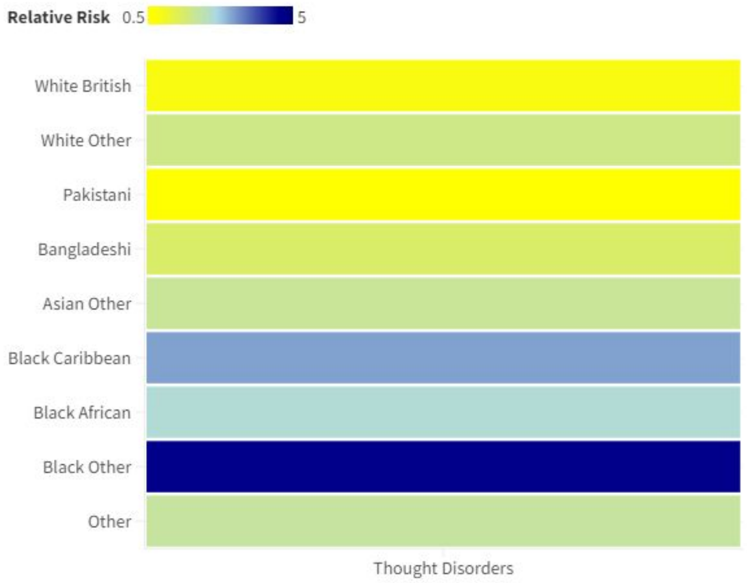

Supplementary Table 12 - Relative Risk Data Summary: Thought Disorders (To accompany Supplementary Figure 4)

Relative Risk (95% CI)	
**White British**	0.85 (0.74-0.97)
**White Other**	1.85 (1.28-2.69)
**Pakistani**	0.72 (0.43-1.21)
**Bangladeshi**	1.59 (0.85-2.97)
**Asian Other**	1.98 (1.21-3.25)
**Black Caribbean**	3.13 (1.88-5.21)
**Black African**	2.50 (1.64-3.82)
**Black Other**	4.57 (3.00-6.97)
**Other**	2.06 (1.33-3.17)
	**Thought Disorders**

Reference group for each outcome (column): All racial-ethnic groups combined.

Table 1. Stress-Related Presentations Codelist

Category		ICD-10 code	ICD-10 Description
(1) Potentially Psycho-Somatic Symptoms – incl. Pain, Cardiovascular/Respiratory, Digestive, Skin, Sleep & Fatigue.			
Potentially Psycho-Somatic Symptoms *	Pain	R10	Abdominal and pelvic pain
	Headache	R51	Headache
		G43	Migraine
		G44	Other headache syndromes
		M54	Panniculitis affecting regions of neck and back
	Other pain	M626	Muscle strain
		M796	Pain in limb
		M255	Pain in joint
		R52	Acute pain
	Circulatory & Respiratory signs	R00	Abnormalities of heartbeat
		R03	Abnormal blood pressure reading, without diagnosis
		R05	Cough
		R06	Abnormalities of breathing
		R07	Pain in throat and chest
	Digestive & Feeding symptoms	R11	Nausea and vomiting
		R12	Heartburn
		R13	Dysphagia
		R14	Flatulence and related conditions
		R63	Symptoms and signs concerning food and fluid intake
		R194	Change in bowel habit
	Skin symptoms	R20	Disturbances of skin sensation
		R21	Rash and other nonspecific skin eruption
		R231	Pallor
		R234	Changes in skin texture
		R238	Other and unspecified skin changes
	Nervous & Musculoskeletal symptoms	R25	Abnormal involuntary movements
		R26	Abnormalities of gait and mobility
		R27	Other lack of co-ordination
		R292	Abnormal reflex
		R293	Abnormal posture

		R294	Clicking hip
		R298	Other and unspecified signs and symptoms involving nervous and musculoskeletal systems
	Cognitive symptoms	R41	Other symptoms and signs involving cognitive functions and awareness
		R42	Dizziness and giddiness
		F05	Delirium, not induced by alcohol and other psychoactive substances
	Malaise, Fatigue & Syncope	R400	Somnolence
		R401	Stupor
		R53	Malaise and fatigue
		R55	Syncope and collapse
	Other/General symptoms	N39.3	Stress incontinence
		R44	Other symptoms and signs involving general sensations and perceptions
		R45	Symptoms and signs involving emotional state
		R46	Symptoms and signs involving appearance and behaviour
		R47	Dysphasia and aphasia
		R49	Voice disturbances
		Z563	Stressful work schedule
		Z564	Discord with boss and workmates
		Z711	Person with feared complaint in whom no diagnosis is made
		Z733	Stress, not elsewhere classified
	Sleep disorders	F51	Nonorganic sleep disorders
		G47	Disorders of initiating and maintaining sleep [insomnias]
* Note: Admissions will not be categorised as potentially psycho-somatic if a medical or surgical cause was indicated by an operation or subsidiary diagnostic code for the same admission.			
Psychopathology – (2) Internalising, (3) Externalising & (4) Thought Disorder Problems/Dysregulation Dimensions			
Internalising	Mood Disorders	F320	Mild depressive episode
		F321	Moderate depressive episode
		F322	Severe depression without psychotic symptoms
		F328	Other depressive episodes
		F329	Depressive episode, unspecified
		F330	Recurrent depressive disorder, current episode mild
		F331	Recurrent depressive disorder, current episode moderate
		F332	Recurrent depressive disorder, current episode severe without psychotic symptoms
		F333	Recurrent depressive disorder, current episode severe with psychotic symptoms
		F338	Other recurrent depressive disorders
		F339	Recurrent depressive disorder, unspecified
		F341	Dysthymia

	Anxiety or fear-related, Obsessive-compulsive, & Dissociative disorders	F40	Phobic anxiety disorders
		F41	Other anxiety disorders
		F94.0	Elective mutism
		F42	Obsessive-compulsive disorder
		F44	Dissociative [conversation] disorders
		F45	Somatoform disorders
		F48	Other neurotic disorders
		F54	Psychological and behavioural factors associated with disorders or diseases classified elsewhere
	Disorders associated with stress or experiences of trauma	F43	Reaction to severe stress, and adjustment disorders
		F62	Enduring personality changes, not attributable to brain damage and disease
		F941	Reactive attachment disorder of childhood
		F942	Disinhibited attachment disorder of childhood
	Eating disorders	F50	Eating disorders
	Emotional disorders with onset usually occurring in childhood and adolescence	F93	Emotional disorders with onset specific to childhood
		F98	Other behavioural and emotional disorders with onset usually occurring in childhood and adolescence
Externalising	Impulse control, Disruptive behaviour or dissocial disorders	F63	Habit and impulse disorders
		F90	Disturbance of activity and attention
		F91	Conduct disorders
		F92	Mixed disorders of conduct and emotions
	Substance use & abuse	F10	Mental and behavioural disorders due to use of alcohol
		F11	Mental and behavioural disorders due to use of opioids
		F12	Mental and behavioural disorders due to use of cannabinoids
		F13	Mental and behavioural disorders due to use of sedatives or hypnotics
		F14	Mental and behavioural disorders due to use of cocaine
		F15	Mental and behavioural disorders due to use of other stimulants, including caffeine
		F16	Mental and behavioural disorders due to use of hallucinogens
		F17	Mental and behavioural disorders due to use of tobacco
		F18	Mental and behavioural disorders due to use of volatile solvents
		F19	Mental and behavioural disorders due to use of multiple drug use and use of other psychoactive substances
		F55	Abuse of non-dependence-producing substances
		R780	Finding of alcohol in blood
		R781	Finding of opiate drug
		R782	Finding of cocaine in blood

		R783	Finding of hallucinogen in blood
		R784	Finding of other drugs of addictive ptential in blood
		R785	Finding of psychotropic drug in blood
		T51	Toxic effect of alcohol
		Y90 [3]	Evidence of alcohol involvement determined by blood alcohol level
		Y91 [3]	Evidence of alcohol involvement determined by level of intoxication
		Z040 [3]	Blood-alcohol and blood-drug test
		Z502 [3]	Alcohol rehabilitation
		Z503 [3]	Drug rehabilitation
		Z714 [3]	Alcohol abuse counselling and surveillance
		Z715 [3]	Drug abuse counselling and surveillance
		Z721 [3]	Alcohol use
		Z722 [3]	Drug use
Thought Disorder Problem	Mood Disorders with mania or psychotic symptoms	F30	Manic episode
		F31	Bipolar affective disorder
		F323	Severe depression with psychotic symptoms
	Schizophrenia or other primary psychotic disorders	F20	Schizophrenia
		F21	Schizotypal disorder
		F22	Persistent delusional disorders
		F23	Acute and transient psychotic disorders
		F24	Induced delusional disorder
		F25	Schizoaffective disorders
		F28	Other nonorganic psychotic disorders
		F29	Unspecified nonorganic psychosis
[3] *Categorised as Externalisng presentation if this ICD-10 code was recorded and one of the following self-harm codes were also recorded in another diagnostic position: X60-63 (Intentional self-poisoning (drugs)), X64-X69 (Intentional self-harm (self-poisoning)), X70-X84 (Intentional self-harm (hanging, drowning, firearm, explosive material, fire, steam, sharp/blunt object, jumping, crashing motor vehicle, other)), Z642 (Intentional self-poisoning by and exposure to other and unspecified drugs, medicaments and biological substances) or Z915 (personal history of self-harm).*			
(5) Self-Harm incl. Non-fatal intentionally self-inflicted physically harmful act, regardless of suicidal intent, i.e., intentional self-injury or self-poisoning			
Self-Harm		T36 [2]	Poisoning by systemic antibiotics
		T37 [2]	Poisoning by other systemic anti-infectives and antiparasitics
		T38 [2]	Poisoning by hormones and their synthetic substitutes and antagonists, not elsewhere classified
		T39 [2]	Poisoning by nonopioid analgesics, antipyretics and antirheumatics
		T40 [2]	Poisoning by narcotics and psychodysleptics [hallucinogens]
		T41 [2]	Poisoning by anaesthetics and therapeutic gases
		T42 [2]	Poisoning by antiepileptic, sedative-hypnotic and antiparkinsonism drugs
		T43 [2]	Poisoning by psychotropic drugs, not elsewhere classified

	T44 [2]	Poisoning by drugs primarily affecting the autonomic nervous system
	T45 [2]	Poisoning by primarily systemic and haematological agents, not elsewhere classified
	T46 [2]	Poisoning by agents primarily affecting the cardiovascular system
	T47 [2]	Poisoning by agents primarily affecting the gastrointestinal system
	T48 [2]	Poisoning by agents primarily acting on smooth and skeletal muscles and the respiratory system
	T49 [2]	Poisoning by topical agents primarily affecting skin and mucous membrane and by ophthalmological, otorhinolaryngological and dental drugs
	T50 [2]	Poisoning by diuretics and other and unspecified drugs, medicaments and biological substances
	Y10 [1]	Poisoning by and exposure to nonopioid analgesics, antipyretics and antirheumatics, undetermined intent
	Y11 [1]	Poisoning by and exposure to antiepileptic, sedative-hypnotic, antiparkinsonism and psychotropic drugs, not elsewhere classified, undetermined intent
	Y12 [1]	Poisoning by and exposure to narcotics and psychodysleptics [hallucinogens], not elsewhere classified, undetermined intent
	Y13 [1]	Poisoning by and exposure to other drugs acting on the autonomic nervous system, undetermined intent
	Y14 [1]	Poisoning by and exposure to other and unspecified drugs, medicaments and biological substances, undetermined intent
	Y15 [1]	Poisoning by and exposure to alcohol, undetermined intent
	Y16 [1]	Poisoning by and exposure to organic solvents and halogenated hydrocarbons and their vapours, undetermined intent
	Y17 [1]	Poisoning by and exposure to carbon monoxide and other gases and vapours, undetermined intent
	Y18 [1]	Poisoning by and exposure to pesticides, undetermined intent
	Y19 [1]	Poisoning by and exposure to other and unspecified chemicals and noxious substances, undetermined intent
	S00 [2]	Superficial injury of scalp
	S10 [2]	Superficial injury of neck
	S20 [2]	Superficial injury of thorax
	S30 [2]	Superficial injury of lower back and pelvis
	S40 [2]	Superficial injury of shoulder and upper arm
	S50 [2]	Superficial injury of forearm
	S60 [2]	Superficial injury of wrist and hand
	S70 [2]	Superficial injury of hip and thigh
	S80 [2]	Superficial injury of lower leg
	S90 [2]	Superficial injury of ankle and foot

	X60[1]	Intentional self-poisoning by and exposure to nonopioid analgesics, antipyretics and antirheumatics
	X61[1]	Intentional self-poisoning by and exposure to antiepileptic, sedative-hypnotic, antiparkinsonism and psychotropic drugs, not elsewhere classified
	X62[1]	Intentional self-poisoning by and exposure to narcotics and psychodysleptics [hallucinogens], not elsewhere classified
	X63[1]	Intentional self-poisoning by and exposure to other drugs acting on the autonomic nervous system
	X64[1]	Intentional self-poisoning by and exposure to other and unspecified drugs, medicaments and biological substances
	X65[1]	Intentional self-poisoning by and exposure to alcohol
	X66[1]	Intentional self-poisoning by and exposure to organic solvents and halogenated hydrocarbons and their vapours
	X67[1]	Intentional self-poisoning by and exposure to carbon monoxide and other gases and vapours
	X68[1]	Intentional self-poisoning by and exposure to pesticides
	X69[1]	Intentional self-poisoning by and exposure to other and unspecified chemicals and noxious substances
	X70[1]	Intentional self-harm by hanging, strangulation and suffocation
	X71[1]	Intentional self-harm by drowning and submersion
	X72[1]	Intentional self-harm by handgun discharge
	X73[1]	Intentional self-harm by rifle, shotgun and larger firearm discharge
	X74[1]	Intentional self-harm by other and unspecified firearm discharge
	X75[1]	Intentional self-harm by explosive material
	X76[1]	Intentional self-harm by smoke, fire and flames
	X77[1]	Intentional self-harm by steam, hot vapours and hot objects
	X78[1]	Intentional self-harm by sharp object
	X79[1]	Intentional self-harm by blunt object
	X80[1]	Intentional self-harm by jumping from a high place
	X81[1]	Intentional self-harm by jumping or lying before moving object
	X82[1]	Intentional self-harm by crashing of motor vehicle
	X83[1]	Intentional self-harm by other specified means
	X84[1]	Intentional self-harm by unspecified means
	Z642 [1]	Seeking and accepting physical, nutritional and chemical interventions known to be hazardous and harmful
	Z915 [1]	Personal history of self-harm

[1] *Categorised as Self-Harm if this ICD-10 code was recorded in any diagnostic coding position.*

[2] Categorised as Self-Harm if this ICD-10 code was recorded in the primary diagnostic coding position and one of the following self-harm codes were also recorded in another diagnostic position: X60-63 (Intentional self-poisoning (drugs)), X64-X69 (Intentional self-harm (self-poisoning)), X70-X84 (Intentional self-harm (hanging, drowning, firearm, explosive material, fire, steam, sharp/blunt object, jumping, crashing motor vehicle, other)), Z642 (Intentional self-poisoning by and exposure to other and unspecified drugs, medicaments and biological substances) or Z915 (personal history of self-harm).

Table 2. Medical & Surgical Exclusion Codes

Category	ICD-10 code	ICD-10 Description
Medical	A00	Cholera
	A01	Typhoid and paratyphoid fevers
	A02	Other salmonella infections
	A03	Shigellosis
	A04	Other bacterial intestinal infections
	A05	Other bacterial foodborne intoxications not elsewhere classified
	A06	Amoebiasis
	A07	Other protozoal intestinal diseases
	A08	Viral and other specified intestinal infections
	A09	Other gastroenteritis and colitis of infectious and unspecified origin
	K520	Gastroenteritis and colitis due to radiation
	K521	Toxic gastroenteritis and colitis
	K529	Noninfective gastroenteritis and colitis unspecified
	J09	Influenza due to identified zoonotic or pandemic influenza virus
	J10	Influenza due to identified seasonal influenza virus
	J13	Pneumonia due to Streptococcus pneumoniae
	J14	Pneumonia due to Haemophilus influenzae
	J15	Bacterial pneumonia not elsewhere classified
	J16	Pneumonia due to other infectious organisms not elsewhere classified
	J17	Pneumonia in diseases classified elsewhere
	J18	Pneumonia organism unspecified
	N81	Female genital prolapse
	N83	Noninflammatory disorders of ovary fallopian tube and broad ligament
	N85	Other noninflammatory disorders of uterus except cervix
	N86	Erosion and ectropion of cervix uteri

	N87	Dysplasia of cervix uteri
	N88	Other noninflammatory disorders of cervix uteri
	N89	Other noninflammatory disorders of vagina
	N90	Other noninflammatory disorders of vulva and perineum
	E282	Polycystic ovarian syndrome
	N31	Neuromuscular dysfunction of bladder not elsewhere classified
	N390	Urinary tract infection site not specified
	K55	Vascular disorders of intestine
	K56	Paralytic ileus and intestinal obstruction without hernia
	G40	Epilepsy
	G41	Status epilepticus
	G45	Transient cerebral ischaemic attacks and related syndromes
	G46	Vascular syndromes of brain in cerebrovascular diseases
	I60	Subarachnoid haemorrhage
	I61	Intracerebral haemorrhage
	I62	Other nontraumatic intracranial haemorrhage
	I63	Cerebral infarction
	I64	Stroke not specified as haemorrhage or infarction
	I65	Occlusion and stenosis of precerebral arteries not resulting in cerebral infarction
	I66	Occlusion and stenosis of cerebral arteries not resulting in cerebral infarction
	I67	Other cerebrovascular diseases
	I68	Cerebrovascular disorders in diseases classified elsewhere
	I69	Sequelae of cerebrovascular disease
	C15	Malignant neoplasms of digestive organs
	C16	Malignant neoplasms of digestive organs
	C17	Malignant neoplasms of digestive organs
	C18	Malignant neoplasms of digestive organs
	C19	Malignant neoplasms of digestive organs
	C20	Malignant neoplasms of digestive organs
	C21	Malignant neoplasms of digestive organs
	C22	Malignant neoplasms of digestive organs
	C23	Malignant neoplasms of digestive organs
	C24	Malignant neoplasms of digestive organs
	C25	Malignant neoplasms of digestive organs
	C26	Malignant neoplasms of digestive organs

	C30	Malignant neoplasms of respiratory and intrathoracic organs
	C31	Malignant neoplasms of respiratory and intrathoracic organs
	C32	Malignant neoplasms of respiratory and intrathoracic organs
	C33	Malignant neoplasms of respiratory and intrathoracic organs
	C34	Malignant neoplasms of respiratory and intrathoracic organs
	C35	Malignant neoplasms of respiratory and intrathoracic organs
	C36	Malignant neoplasms of respiratory and intrathoracic organs
	C37	Malignant neoplasms of respiratory and intrathoracic organs
	C38	Malignant neoplasms of respiratory and intrathoracic organs
	C39	Malignant neoplasms of respiratory and intrathoracic organs
	C40	Malignant neoplasms of bone and articular cartilage
	C41	Malignant neoplasms of bone and articular cartilage
	C45	Malignant neoplasms of mesothelial and soft tissue
	C46	Malignant neoplasms of mesothelial and soft tissue
	C47	Malignant neoplasms of mesothelial and soft tissue
	C48	Malignant neoplasms of mesothelial and soft tissue
	C49	Malignant neoplasms of mesothelial and soft tissue
	C51	Malignant neoplasms of female genital organs
	C52	Malignant neoplasms of female genital organs
	C53	Malignant neoplasms of female genital organs
	C54	Malignant neoplasms of female genital organs
	C55	Malignant neoplasms of female genital organs
	C56	Malignant neoplasms of female genital organs
	C57	Malignant neoplasms of female genital organs
	C58	Malignant neoplasms of female genital organs
	C64	Malignant neoplasms of urinary tract
	C65	Malignant neoplasms of urinary tract
	C66	Malignant neoplasms of urinary tract
	C67	Malignant neoplasms of urinary tract
	C68	Malignant neoplasms of urinary tract
	C69	Malignant neoplasms of eye brain and other parts of central nervous system
	C70	Malignant neoplasms of eye brain and other parts of central nervous system
	C71	Malignant neoplasms of eye brain and other parts of central nervous system
	C72	Malignant neoplasms of eye brain and other parts of central nervous system
	C73	Malignant neoplasms of thyroid and other endocrine glands

	C74	Malignant neoplasms of thyroid and other endocrine glands
	C75	Malignant neoplasms of thyroid and other endocrine glands
	K35	Diseases of appendix
	K36	Diseases of appendix
	K37	Diseases of appendix
	K38	Diseases of appendix
Surgical	Y752	Laparoscopic approach to the abdominal cavity not elsewhere classified
	H013	Emergency excision of normal appendix
	H029	Unspecified excision of appendix
	H012	Emergency excision of normal appendix not elsewhere classified
	H019	Unspecified emergency excision of appendix
	H021	Interval appendectomy
	H023	Prophylactic appendectomy NEC
	H024	Incidental appendectomy
	H028	Other specified excision of appendix
	H011	Emergency excision of abnormal appendix and drainage HFQ
